# Comprehensive Characterization of Multitissue Expression Landscape, Co-Expression Networks and Positive Selection in Pikeperch

**DOI:** 10.3390/cells10092289

**Published:** 2021-09-02

**Authors:** Julien Alban Nguinkal, Marieke Verleih, Lidia de los Ríos-Pérez, Ronald Marco Brunner, Arne Sahm, Saptarshi Bej, Alexander Rebl, Tom Goldammer

**Affiliations:** 1 Institute of Genome Biology, Research Institute for Farm Animals (FBN), 18196 Dummerstorf, Germany; verleih@fbn-dummerstorf.de (M.V.); brunner@fbn-dummerstorf.de (R.M.B.); rebl@fbn-dummerstorf.de (A.R.); 2 Institute of Genetics and Biometry, Research Institute for Farm Animals (FBN), 18196 Dummerstorf, Germany; perez@fbn-dummerstorf.de; 3 Computational Biology of Aging, Fritz Lipmann Institute (FLI)-Leibniz Institute on Aging, 07745 Jena, Germany; arne.sahm@leibniz-fli.de; 4 Department of Systems Biology and Bioinformatics, Faculty of Computer Science and Electrical Engineering, University of Rostock, 18057 Rostock, Germany; saptarshi.bej@uni-rostock.de; 5 Faculty of Agriculture and Environmental Sciences, University of Rostock, 18059 Rostock, Germany

**Keywords:** RNA-Seq, tissue-specificity, positive selection, fish transcriptomics, co-expression

## Abstract

Promising efforts are ongoing to extend genomics resources for pikeperch (*Sander lucioperca*), a species of high interest for the sustainable European aquaculture sector. Although previous work, including reference genome assembly, transcriptome sequence, and single-nucleotide polymorphism genotyping, added a great wealth of genomic tools, a comprehensive characterization of gene expression across major tissues in pikeperch still remains an unmet research need. Here, we used deep RNA-Sequencing of ten vital tissues collected in eight animals to build a high-confident and annotated trancriptome atlas, to detect the tissue-specificity of gene expression and co-expression network modules, and to investigate genome-wide selective signatures in the Percidae fish family. Pathway enrichment and protein–protein interaction network analyses were performed to characterize the unique biological functions of tissue-specific genes and co-expression modules. We detected strong functional correlations and similarities of tissues with respect to their expression patterns—but also significant differences in the complexity and composition of their transcriptomes. Moreover, functional analyses revealed that tissue-specific genes essentially play key roles in the specific physiological functions of the respective tissues. Identified network modules were also functionally coherent with tissues’ main physiological functions. Although tissue specificity was not associated with positive selection, several genes under selection were found to be involved in hypoxia, immunity, and gene regulation processes, that are crucial for fish adaption and welfare. Overall, these new resources and insights will not only enhance the understanding of mechanisms of organ biology in pikeperch, but also complement the amount of genomic resources for this commercial species.

## 1. Introduction

Pikeperch (*Sander lucioperca*), a member of the *Percidae* family, is a fresh and brackish water fish widely distributed in Eurasia. The European project *DIVERSIFY* [1,2] has identified pikeperch as one of the six species with the highest potential for inland aquaculture diversification in Europe [3,4]. This perch-like species emerged as an attractive candidate for aquaculture development, especially due to its flesh quality, which displays low fat content, highly assimilable proteins, and delicate flavour without small intramuscular bones. This makes pikeperch a highly demanded and expensive product in the international markets. Moreover, its relatively rapid growth and resilience to disease and handling stress in captive environments, compared to other percids [2,5], make pikeperch an interesting species for intensive aquaculture systems. However, a number of production issues remain unresolved. These include juvenile cannibalism, low larval survival, high incidence of deformities, impaired and nonuniform growth [6,7,8,9].

Due to the rapid development of next-generation sequencing (NGS) technology, significant efforts have been made in recent years to provide genomics resources for pikeperch, resulting in a significant amount of multi-omics studies and data. In fact, more than 90% of multi-omics studies in pikeperch, as recorded by PubMed Central, 30 June 2021, have been published in the last five years (2016–2021). Notable genomics efforts include the transcriptome profiling of eggs from wild and domesticated populations along with the generation of the first transcriptomic resource for pikeperch [10], the development of transcriptome-based simple sequence repeats (SSR) markers [11], and the construction of high-density linkage maps using specific locus amplified fragment sequencing (SLAF-Seq) [12], and single-nucleotide polymorphism (SNPs) markers [13]. The recent publication of the chromosome-level reference genome (SLUC_FBN_1.2) for *S. lucioperca* [14] has dramatically improved the ability to investigate its complex traits with commercial relevance. Much more, it has paved the way for further genomic studies such as genotyping by sequencing and genetic markers identification, essential to improve the biological efficiency of aquaculture production traits through selective breeding.

Tissue-specific genes are those with significantly enhanced expression levels in a given tissue, relative to the baseline expression in all other tissues. Tissue-specific expression profiling is crucial in elucidating the development, the complexity, and evolutionary history of an organism at the systemic level. Furthermore, the classification of genes with regard to their expression patterns across organs or tissue types is important for a deeper understanding of the molecular mechanisms of tissue activity and function, to discover key regulatory features, and to shed light on the correlated phenotypic and functional evolution of tissues [15,16,17]. Tissue-specific expression profiles have been characterized in different aquaculture species, including crucian carp (*Carassius carassius*) [18], Atlantic salmon (*Salmo salar*) [19], and rainbow trout (*Oncorhynchus mykiss*) [20]. In Atlantic salmon for example, analysing genes uniquely expressed in brain-pituitary-gonad tissues has provided a basic understanding of key production and life history traits in salmonids [19]. So far, no comprehensive tissue-specific gene expression atlas of pikeperch has been characterized, and similar analyses have not been performed for any other Percid species. This work aims to fill that gap with a comprehensive catalog of tissue-specific protein-coding genes expressed in pikeperch using deep multitissue RNA sequencing (RNA-Seq).

Spatial transcriptomics (e.g., multitissue) can enable to explore the adaptive evolution of genes, i.e., genes under positive selection (GUPS), and provide a foundation for co-expression network analyses across tissues. Actually, GUPS can point out lineage-specific evolutionary and adaptation features among species, while analysing co-expression networks can reveal the functional property of genes under different biological and cellular processes, because genes sharing similar functions will tend to be co-expressed across cell types or tissues [21]. Another interesting question is whether the expression of genes evolving under positive selection in pikeperch is correlated with tissue specificity, or if in addition, GUPS act as hubs (key players) in co-expression network modules. To our knowledge, no study has yet characterized the co-expression modules and networks from multiple tissues in pikeperch.

To address the questions above, we report in this study, a new functionally annotated high-quality multitissues transcriptome assembly, genome-wide tissue-specific expression atlas and functional co-expression networks in pikeperch. As an initial application of this new genomic resource, we investigated the evolutionary signatures in six representative percids species and clarified that protein evolution in pikeperch is not correlated with tissue-specificity. To capture the variety of tissue-specific processes and better characterize the tissue function, we identified candidate pathways and biological processes associated with tissue-specific genes and co-expression modules in this aquaculture species.

## 2. Materials and Methods

All procedures involving the handling and treatment of fish used in this study were approved by the Committee on the Ethics of Animal Experiments of Mecklenburg-Western Pomerania (Landesamt für Gesundheit und Soziales LAGuS, Approval ID: 7221.3-1-009/19).

### 2.1. Tissue Sampling, Library Preparation, RNA-Sequencing

Tissue samples were collected from eight adult pikeperch individuals (3 males, 5 females) in the Experimental Aquaculture Facility of the Research Institute for Farm Animal Biology (FBN). Prior to tissue collection, fish were euthanized by immersion for 15 min in an overdose of 2-phenoxyethanol (50 mg/L) followed by a bleed cut in the head as well as cutting of the spinal cord posterior to the head. For each individual, different tissues including gonads (testis or ovary), liver, spleen, muscle, gills, brain, head kidney, skin, and heart were sampled and snap-frozen in liquid nitrogen. They were ultimately transferred to a −80 ${}^{{}^{\circ}}$C freezer until required for RNA extraction.

In total, eight individuals were euthanized and 72 tissue samples were obtained (Supplementary file S1). These tissue samples were separately homogenized in 1 mL TRIzol reagent (Invitrogen, Darmstadt, Germany). Following phenol-chloroform extraction, the obtained RNA was purified using the RNeasy Mini Kit (Quiagen, Hilden, Germany) according to the manufacturer’s protocol. Extracted RNA was quantified using the NanoDrop (Thermo Scientific™ NanoDrop 2000) and its integrity was assessed by electrophoretic profiling with Agilent Bioanalyzer 2100 (Agilent, Santa Clara, CA, USA). We subsequently pooled the purified mRNA from the same tissue type. With exception of the gonads sample which are (clearly) sex homogeneous, the other pools are sex heterogeneous in the ratio of 3 males vs. 5 females (Supplementary file S1).

Finally, we prepared two equimolar aliquots of 25 μL for each pool, to obtain duplicates of each tissue sample for library preparation, where we used polyA selection protocol coupled with TruSeq Stranded mRNA LT Sample Prep Kit v2 (Illumina, San Diego, CA, USA), following the manufacturer’s standard protocol. Stranded-specific (forward) paired-end 150 bp reads (PE150) were generated with the NovaSeq 6000 System. In total, 18 PE150 libraries were sequenced (2 × 8 non-gonadal tissues + 2 × gonads) (Table 1). We ultimately performed quality control (QC) including adapters removal and screening for contamination using the fastp pipeline (version 0.20.1) [22], whereby low quality reads (<Q25) were discarded.

### 2.2. De Novo Transcriptome Assembly, Functional Annotation

To achieve the most biologically meaningful and representative set of *S. lucioperca* transcripts, we used a combination of different assembly strategies. De novo assembly algorithms included Trinity (version 2.8.1) [23], and rnaSPAdes (version 3.14.1) [24]. The Trinity assembly was performed by pooling reads of all tissues and setting strand-specific parameters. The rnaSPAdes assembly was iteratively built with k-mer sizes of 27, 33, 55, 77, and 99. Additionally, we generated a genome-guided assembly with StringTie2 (version 2.1.2) [25]. Briefly, the combined RNA-Seq reads of all tissues were aligned to the latest pikeperch reference genome (SLUC_FBN_1.2, GenBank accession: GCA_008315115.2) [13] using HISAT2 (version 2.2.0) [26]. Reads alignments were then assembled with StringTie2 [25] using the ‘−merg’ option to obtain a non-redundant set of transcripts across all tissue samples. Subsequently, raw assemblies were piled into a meta-assembly and large redundant transcripts were clustered using cd-hit-est (version 4.6.1) [27] with an identity threshold of 98%. Note that our clustering threshold of 98% is slightly lower than the 100% suggested by Nakasugi et al. [28]. This approach was chosen because the pooled libraries of our tissue samples represent individuals with different genotypes. A too stringent identity threshold (e.g., 100%), would result in too many transcript variants remaining unclustered, and thus make the redundancy removal suboptimal. Finally, we used EvidentialGene tr2aacds pipeline [29] to collate the overassemblies into a less redundant and high confident transcript set, thereby maximizing the diversity and completeness of the final transcriptome assembly. In brief, EvidentialGene pipeline takes a pile of transcripts from different assemblies, predicts amino acid and transcript coding potential (1), removes redundant fragments and clusters highly similar fragments into transcript loci (2), picks the “best” non-redundant mRNA for each loci, and ultimately classifies its transcripts as “primary” or “alternative” (3). Unless stated otherwise, only primary transcripts were used for quality assessment, functional annotation, and further downstream analyses.

The resulted multitissue transcriptome was functionally annotated using the eggNOG mapper (version 5.0) [30] as well as through homology search against protein sequence databases, including SwissProt and nonredundant RefSeq (NCBI) proteins. Moreover, we performed protein domain identification and functional sites mapping with InterProScan (version 5) [31].

### 2.3. Quality Assessment of the Multitissues Transcriptome Assembly

The quality of our newly built transcriptome assembly was gauged using multiple strategies and quality metrics. Assembled transcripts were aligned with minimap2-splice option (version 2.21) [32] to the reference genome (GCF_008315115.2) to produce a GTF annotation file which was then compared to the pikeperch reference annotation (GCF_008315115.2) with gffcompare (version 0.10.4) [33]. We also used BUSCO (version 4.1.4) [34] to explore the assembly completeness regarding the conserved actinopterygians (Actinopterygii dataset) single-copy orthologs. To assess the RNA-Seq reads representation of the assemblies, combined RNA-Seq reads were mapped to the assembly using Hisat2, and the mapping statistics was estimated. Finally, we searched (BLAST) the assembled transcripts against protein databases including UniProt and NCBI RefSeq nonredundant proteins, and performed full length transcript analysis using an utility Perl script provided in Trinity.

### 2.4. Quantification of Tissues Expression Profiles

The trimmed and filtered RNA-Seq reads from each sample were individually mapped to the pikeperch reference genome (SLUC_FBN_1.2) [13] using STAR (version 2.7.5a) [35], in two-pass mode. Abundance levels of transcripts were estimated using TPMCalculator [36], a one-step software to quantify mRNA expression abundance directly from RNA-Seq alignments. TPMCalculator reported the expression matrix including transcripts per million (TPM) values and raw read counts for each gene across all samples. We removed genes with mean expression over all tissues ≤1 TPM, as well as those tagged as noncoding RNA (ncRNA).

### 2.5. Tissue Specificity Index, Differential Expression Analysis

The per sample average (arithmetic mean) expression values (TPM) were used to calculate the index of tissue specificity (τ) for all pikeperch protein-coding genes. Following the approach described in Yanai et al. [37] and Mank et al. [38], we calculated tissue specificity (τ) for protein-coding genes with the formula:

$$\tau =\frac{\sum_{i=1}^{N}\left(1-\frac{TPM_{g,i}}{TPM_{g,max}}\right)}{N-1}$$

where *N* is the total number of tissues examined, $TPM_{g,i}$ is the expression of a gene *g* in tissue *i*, and $TPM_{g,max}$ is the maximal expression level detected for a given gene *g* over the examined *N* tissues. Tau (τ) index has been demonstrated to be the most suitable metric to measure gene tissue specificity [39]. It varies between 0 and 1, where highly tissue-specific transcripts have values approaching 1 (τ>0.85), and broadly expressed transcripts (e.g., housekeeping genes) have a tissue-specificity index approaching 0 (τ<0.3) [17,39,40]. In addition, we analysed differential expression (DE) between tissue samples in a “one vs. all” design, utilizing the likelihood ratio testing (LRT) under the generalized linear model (GLM) framework in the package edgeR [41]. We have iteratively detected genes whose expression levels change by a significant amount between the two groups—namely *X* and non-*X*, where X is one of our 10 tissue samples. Instead of considering only statistical significance like in standard DE tests, we applied a combination of fold-change ($\log_{2}FC>3$) and *p*-value (p<0.05) cut-offs to deem genes as differentially expressed (DEGs) between the respective comparison groups (*X* vs. non-*X*).

Lastly, we performed tissue enrichment analysis using the *teGeneRetrieval* function in the Bioconductor package TissueEnrich [42]. This package applies the algorithm from the Human Protein Atlas (HPA) [43] on expression data (normalized counts) and classifies genes into different categories based on their expression levels across the tissues. More details about the tissueEnrich algorithm can be found in Jain et. al [42].

### 2.6. Tissue-Specific Co-Expression and Network Modules Analysis

To assess the tissue-specificity of genes in the context of functional modules, we examined whether tissue-specific expression patterns are also reflected in tissue-specific co-expression and network modules. To that end, we used CEMiTool [44], a Bioconductor package to identify differential co-expression modules. Tissue-specific co-expression modules were defined as a subset of tissue-specific genes which show relatively high co-expression in one tissue, while having consistently lower co-expression in all other tissues. Tissue-specific protein–protein interaction (PPI) networks were predicted as follows: we first constructed global (i.e., based on all genes) interactions by exhaustively mapping pikeperch proteins to PPI networks supported by experimental evidences using STRING (version 11). Only proteins with a minimum interaction score of more than 0.7 were kept in the PPI network. Given a pikeperch tissue, a subnetwork in the global network is labeled specific to that tissue if the interacting proteins in that subnetwork are differentially co-expressed and tissue-specific. Hence, a PPI network is specific to a given tissue, if it is induced by tissue-specific proteins, whose coding genes are additionally co-expressed in that tissue.

### 2.7. Positive Selection Analysis

We identified GUPS in pikeperch to examine how recent natural selection might be associated with tissue specificity, and to interrogate how it might have shaped the phenotypic and physiological diversification in the *Percidae* branch. To that end, we performed genome-wide analysis of positive selection in six representative species in the *Percidae* family. Briefly, we obtained coding sequences of six percids species, including pikeperch, walleye (*Sander vitreus*; GenBank-Accession: GCA_009193085.1), yellow perch (*Perca flavescens*; GenBank-Accession: GCA_004354835.1) [45], European perch (*Perca fluviatilis*; GenBank-Accession: GCA_010015445.1), Arkansas darter (*Etheostoma cragini*; GenBank-Accession: GCA_013103735.1), and orangethroat darter (*Etheostoma spectabile*; GenBank-Acession: GCA_008692095.1) [46]. One-to-one single-copy orthologs between these species were predicted using OrthoFinder [47]. Based on these single-copy orthologs, positive selection was scanned using PosiGene pipeline [48], which makes use of CODEML program in the PAML package to conduct branch-site tests of positive selection. Yellow perch was used as anchor species, while the Asian sea bass (*Lates calcarifer*), channel bull blenny (*Cottoperca gobio*), and giant grouper (*Epinephelus lanceolatus*) were used as outgroup species in the analysis. Candidate genes under positive selection were those with a false discovery rate (FDR) < 0.1. Finally, we explored the relationship between positive selection, tissue specificity, and gene expression levels.

### 2.8. Functional Enrichment Analyses

To gain insights into the functions, biological processes and pathways associated with GUPS, tissue-specific genes and tissues specific network modules, we performed GO and pathway enrichment analyses using g:Profiler [49]. Furthermore, we assessed biological processes, molecular functions and KEGG pathways involving GUPS and non-GUPS (i.e., all pikeperch genes that are likely not under positive selection). GO categories and KEGG pathways with a FDR ≤0.05 were considered significant. Though only significant terms and pathways associated with at least 3 genes were ultimately retained.

## 3. Results

### 3.1. RNA-Seq, Assembly and Functional Annotation

**Figure 1:**
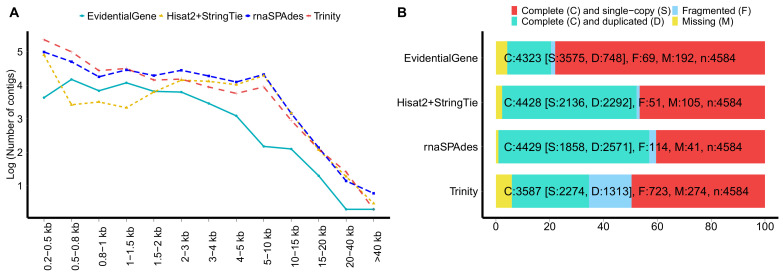
Contigs length distribution and assessment of transcriptomes completeness as determined by Benchmarking Universal Single-Copy Orthologous (BUSCO). (**A**), Contigs length (scaled to log10) of the different transcriptome assemblies. (**B**), BUSCO completeness for each assembly, showing the proportion (%) of complete (C) and single-copy orthologs (S), complete and duplicated (D) orthologs, missing (M) and fragmented (F) orthologs. Transcripts were queried against the Actinopterygii gene set (N = 4584).

The transcriptome profiles of ten different pikeperch tissues were analysed using RNA-Seq methods. Messenger RNA Sequencing yielded between 32.5 and 49 million PE-reads per library, with an average of nearly 38 million PE-reads (Table 1). About 92% of the raw reads were ultimately retained after QC. Different sets of transcriptome assemblies, including Trinity, rnaSPAdes, and Stringtie2 were then built. A summary of the assemblies statistics and their characteristics including functional annotation, are reported in Table 2 and Figure 1. The number of transcripts greatly varies among assemblies. De novo assembly with Trinity and rnaSPAdes substantially yielded a larger number of contigs, with 438,462 and 295,387 contigs, respectively. The reference-guided assembly with StringTie2 yielded 79,936 contigs in total. As expected, merging all assemblies with EvidentialGene substantially reduced the contig count to a number of 56,302 contigs, which is quite consistent with the total number of proteins (N = 56,557) annotated in the pikeperch reference genome [13], indicating that our multitissue-assembly nearly spans the whole pikeperch proteome. Overall, the meta-transcriptome outperformed the separate assemblies (Trinity, rnaSPAdes and Hisat2+StringTie2) in terms of BUSCO completeness and protein functional database records. Interestingly, mapping our transcriptome assembly to the pikeperch reference genome showed that all reference loci were recovered (100%), and approx. 94% of reference introns were accurately captured, while only 4% of reference exons were missed by our assembly, validating the high accuracy of our transcriptome assembly. Regarding assembly metrics and transcript coverage, Hisat2+StringTie2 yielded the best results (Table 2, Figure 1A). In particular, nearly 89% of transcripts were assembled in full length (Table 2). This result is in line with previous studies [50], where reference-guided assemblies tended to produce longer and more full-length transcripts compared to reference-free approaches.

### 3.2. Expression Atlas of Pikeperch Protein-Coding Genes

Most of the expressed putative protein-coding genes were detected in the testis (N = 22,097), brain (N = 19,481) and gills (N = 17,417), while muscle expressed the least genes (N = 10,529). A mean number of 15,820 genes were detected per tissue. Since cDNA libraries were constructed with equal amounts of cDNA from each tissue, the differences in the number of detected protein-coding genes suggest genuine biological variations. We classified all protein-coding with detected expression signals (N = 19,542) into 4 main categories, according to their expression levels and tissue-specificity index (τ) (Table 3, Supplementary file S2).

#### 3.2.1. Mixed-Expressed Genes

**Figure 2:**
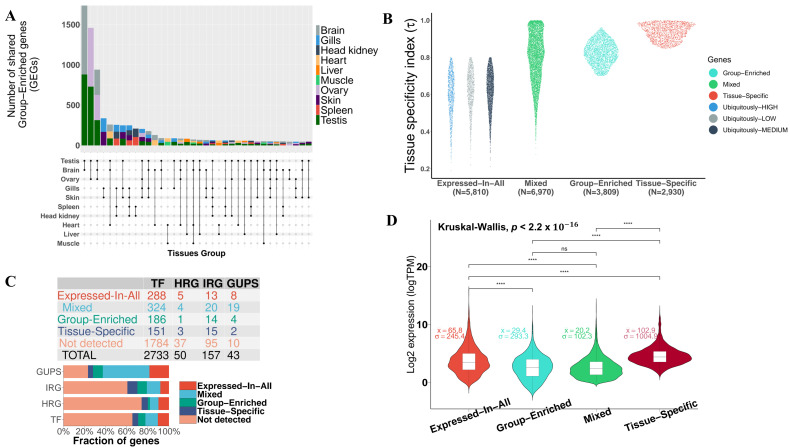
Classification of protein-coding genes detected in pikeperch (*Sander lucioperca*). (**A**), Upset plot depicting different gene sets and the number of shared Group-Enriched genes in each set. (**B**), Sinaplot showing the distribution of the tissue specificity index τ in each genes class. (**C**), Number of transcription factors (TF), hypoxya-related genes (HRG), immune-related genes (IRG), and genes under positive selection (GUPS) in *S. lucioperca*, found in each category. (**D**), Violin plot showing the distribution of genes expression levels withing each class. Statistical significance ns: Not significant; ****: Extremely significant (p<0.0001).

The largest class of genes (35.7%) consisted of 6970 genes in the category termed “Mixed”, which includes detected genes that could not be assigned to any of “Tissue-Specific”, “Group-Enriched” or “Expressed-In-All” categories (Figure 2). This class showed the lowest expression variance (σ=102.3) and least average expression ($\bar{x}=20.2$), suggesting a lower within-group expression variability. Moreover, the τ index in this category is more dispersed (σ=0.13) compared to the three other categories (σ<0.06). This is coherent with our definition of *Mixed-Expressed* genes, which are highly enriched in a subset of tissues while being broadly expressed at moderate or lower levels in the others. Thus, this explains the stretched distribution of the tissue specificity index (Figure 2B). GO enrichment analysis revealed that 3,586 genes (51.44% of “Mixed-Expressed”) contained significant (FDR<0.05) enrichment for 78 GO-terms (20 GO:MF, 44 GO:BP, 14 GO:CC), five KEGG and two Reactome (REAC) pathways. However, most of these genes (>80%) were associated with only two significant GO terms, namely GO:0005515 (MF:Protein binding, FDR < $10^{-9}$) and GO:0003824 (MF:Catalytic activity, FDR < $10^{-4}$). Transcription factor genes (TF) of which 2733 have been identified in fish species and reported in the Animal Transcription Factor Database (AnimalTFDB3.0) [51], were mostly (12% of all TF genes in fish) found in this category. The fraction and number of TF genes classified in each category are depicted in Figure 2C.

#### 3.2.2. Expressed-in-All Genes

**Figure 3:**
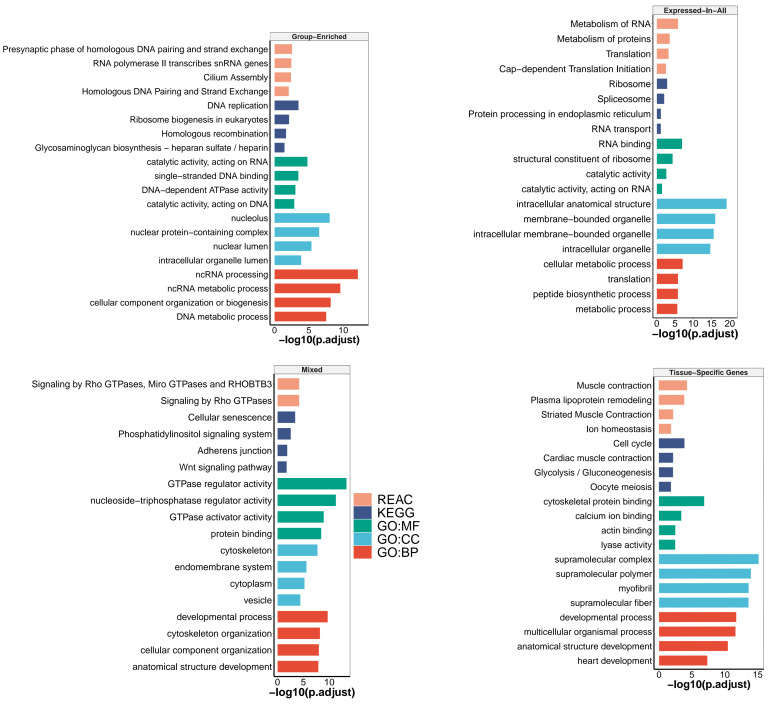
Enrichment of Gene Ontology (GO) terms, KEGG and Reactome (REAC) pathways for each genes class. The top 4 significant (FDR < 0.05) GO terms/functional pathways are depicted here, including REAC (Reactome Pathways), KEGG (KEGG Pathways), GO:BP (Biological Process), GO:CC (Cellular Component), and GO:MF (Molecular Function).

The second largest class (29.8%) consists of 5810 genes ubiquitously expressed in all tissues, termed “Expressed-In-All”. These gene products are needed in all cells and tissues for the maintenance of essential cellular functions. Evidences of functional enrichment (GO, KEGG, REAC) of these genes include primarily ribosomal and spliceosomal proteins involved in protein biosynthesis and metabolism, RNA processing and transport, as well as proteins responsible for the structural integrity and stability of the cell (Figure 3). The average expression level ($\bar{x}=65.8$) in this group is significantly higher (*p*-value < $10^{-16}$) than in “Mixed-Expressed” and “Group-Enriched genes”, but still lower than in “Tissue-Specific” genes ($\bar{x}=102.9$), suggesting that these genes are relatively upregulated throughout all analysed pikeperch tissues (Figure 2D). The top 5 most abundant “Expressed-In-All” genes include known housekeeping genes, such as *EEF1A1* (Elongation factor 1-alpha 1), *ACTB2* (Beta-actin), *RPS2* (40S ribosomal protein S2), *RPL7A* (60S ribosomal protein L7a) and *RPL4* (60S ribosomal protein L4).

#### 3.2.3. Group-Enriched Genes

The third category contains 3809 genes (19.5%), termed “Group-Enriched”. Group-Enriched genes are non-housekeeping genes with enhanced expression in a limited number of 2–7 tissues and with an index of tissue specificity τ>0.5. They are often involved in coordinated biological processes in different tissues/organs, and thus highly enriched in those tissues. We obtained 36 sets of *Group-Enriched* genes, comprising two (12 sets) to five (one set) different tissues. The groups {brain; testis} (N = 1760) and {ovary; testis} (N = 1458) are the pairs sharing most of the Group-Enriched genes (Figure 2A). GO overrepresentation analysis indicated that genes enriched in the group {brain; testis} are predominantly involved in plasma membrane bounded cell projection organization (GO:0120036; FDR < $10^{-16}$), and small conductance calcium-activated potassium channel activity (GO:0016286; FDR < $10^{-3}$). Genes in the group {ovary; testis} (gonads) are primarily involved in cellular nitrogen compound metabolism (GO:0034641; FDR = 0) and in nucleotide and nucleic acid metabolic process (GO:0006139; FDR = 0). Genes enriched in the triplet {brain; testis; ovary} did not have any overrepresented GO terms. Global functional analyses indicated that ncRNA processing (GO:0034660) was the most significant biological process of all Group-Enriched genes (Figure 3).

#### 3.2.4. Tissue-Specific Genes

**Figure 4:**
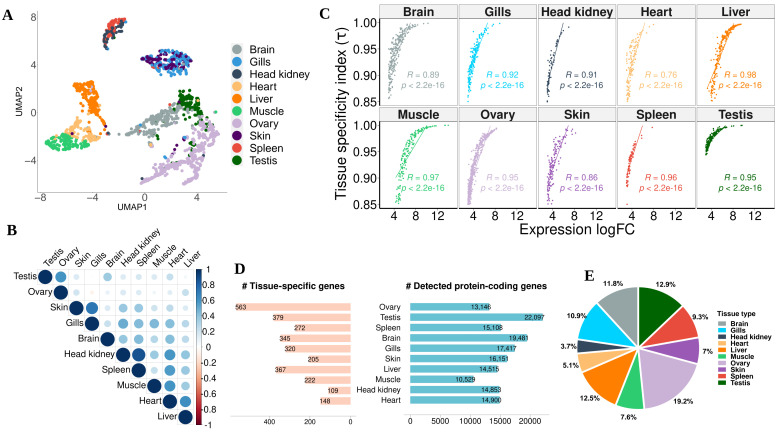
Statistics on tissue-specific genes of *Sander lucioperca*. (**A**), Uniform Manifold Approximation and Projection (UMAP) clustering of 2930 tissue-specific genes in pikeperch based on their expression levels (TPM), where clusters represent genes with similar or correlated expression. (**B**), Correlation heatmap between tissues, based on their specific transcriptome profile. (**C**), Spearman correlation between expression fold-change for each tissue vs. all, and the index of tissue specificity (τ). *R* is the spearman correlation coefficient and *p* the corresponding *p*-value. (**D**), Number of detected and tissue-specific genes in each tissues. (**E**), Percentage distribution of tissue-specific genes across tissues.

The last category termed “Tissue-Specific” (N = 2930) constitutes about 15% of all detected protein-coding genes in pikeperch (Table 3). These are genes with an index of tissue-specificity τ>0.85 and at least five-fold higher expression in one tissue compared to all other tissues. Ovary (N = 563) and testis (N = 379) had the largest numbers of tissue-specific genes detected in our analysis, while the head kidney (N = 109) had the least (Figure 4D). GO enrichment analyses indicated that the most significant biological process in the ovary is cell cycle process (GO:0022402), reproductive process (GO:0022414) in the testis, homeostasis (GO:0007599) in liver, nervous system development (GO:0007399) in brain, developmental process (GO:0032502) in gills, humoral immune response (GO:0006959) in spleen, muscle structure development (GO:0061061) in muscle, circulatory system development (GO:0072359) in heart, and hematopoietic stem cell migration (GO:0035701) in head kidney. Tissue-specific genes in the skin did not show any significantly overrepresented terms. Moreover, KEGG pathway analysis was performed to identify which pathways were significantly enriched with tissue-specific genes. A total of nine significantly enriched pathways were identified, whereas cell cycle, biosynthesis of antibiotics, glycolysis/gluconeogenesis and biosynthesis of amino acids showed the strongest KEGG enrichment signal across multiple tissues. For example, seven tissue types were involved in the biosynthesis of amino acids with at least two genes (Supplementary Figure S1). As expected by construction, the logarithmized expression fold-change for a gene in a given tissue compared to all others tissues is positively correlated with the index of tissue specificity (τ), confirming that tissue-specific genes are significantly upregulated only in a particular tissue (Figure 4C).

We investigated the correlation and similarities between tissues by Uniform Manifold Approximation and Projection (UMAP) clustering of tissues-specific genes based on their expression profiles (TPM) across tissues. We additionally computed the pairwise correlation matrix between all tissues based on the transcript expression levels of tissue-specific genes (N = 2930). UMAP clustering did not only reveal the relationship and similarity between different tissues, but also confirmed the uniqueness of these tissue-specific genes. For example, head kidney and spleen, which are the major lymphoid organs in teleosts, formed a single heterogeneous cluster, suggesting high gene relationships between these tissues. Another similarity of transcriptional profiles was observed for gills and skin tissues, which play an important role in the fish’s physiological exchange between the internal and external environment, and in the regulation of its osmotic pressure. Although genes specific to other tissues, including heart, muscle, liver, brain, testis, and ovary formed distinct and nearly homogeneous clusters, the global inter-cluster relationship was minimized for tissue types involved in coordinated biological processes and sharing common features. For instance, striated muscle tissues including muscle and heart tissues are projected in the same manifold—the liver, which is known to be part of coordinated metabolic activity with skeletal muscle, is clustered in the same manifold with muscle and heart. This discrete grouping of tissue-specific genes is verified by the correlation heatmap highlighting strong correlation between tissues with similar or coordinated biological functions (Figure 4B).

### 3.3. Co-Expression Modules, Hubs and Tissue-Specific Networks

**Figure 5:**
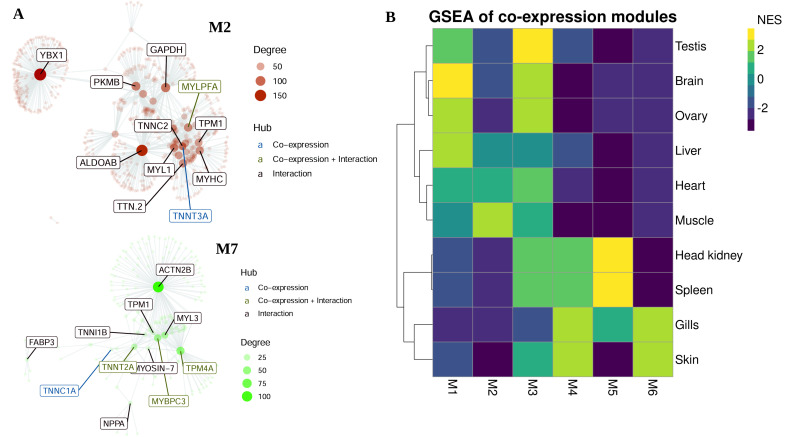
Gene co-expression network analysis. (**A**), Gene co-expression networks of modules M2 (upregulated in muscle tissues) and M7 (upregulated in heart tissues). The top hubs (i.e., genes with highest connectivity) are labelled and coloured based on their source: if only present in the co-expression module predicted by CEMiTool, they are coloured blue; if additionally present in PPI networks, they are coloured green; if exclusively present in PPI network and not in co-expression a network, they are coloured red. The size of the node is proportional to its degree. (**B**), Gene Set Enrichment Analyses (GSA) showing the modules activity on each tissues type. NES is the normalized enrichment score. Exhaustive figures for co-expression modules are available in Supplementary file S4.

To gain insights into the pikeperch interactome with the aim to detect hubs and co-expression networks containing tissue-specific genes, we conducted genome-wide co-expression network analysis. Overall, we identified seven differentially co-expressed modules (M) displaying significantly correlated expression, ranging from 55 to 14 genes in size, and involving 211 genes in total. The largest modules consisted of 55 (M1) and 53 (M2) genes. They were specifically upregulated in liver and muscle tissues, respectively (Table 4). By integrating interactome information with co-expression modules, we identified potential hub genes (i.e., genes that have a high degree of intramodule connectivity) specific to each module (Figure 5A). Gene set enrichment analysis (GSEA) highlighted which modules were induced or repressed in the different tissues (Figure 5B). Finally, we performed overrepresentation analysis (ORA) to determine which biological functions are associated with the identified modules. For instance, the glycolysis/gluconeogenesis pathway is overrepresented in module M4, which is enriched by muscle-specific genes (Supplementary file S4).

**Figure 6:**
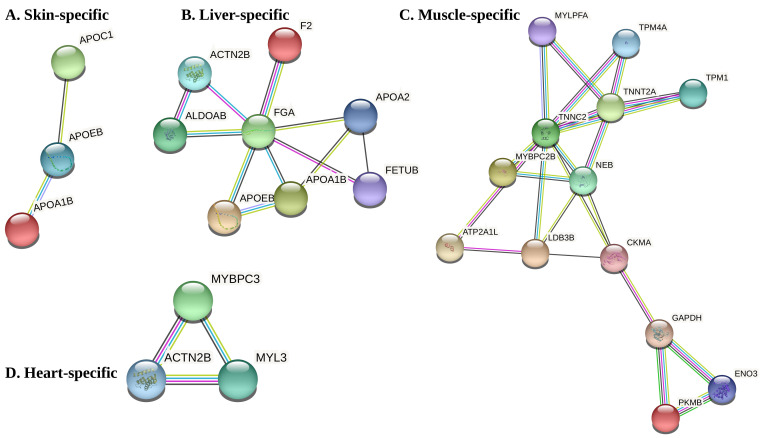
Tissue specific protein–protein interaction networks predicted in four pikeperch tissues including skin, liver, muscle and heart.

To identify network-based protein functional modules that are significantly associated with different tissues, we combined tissue specific co-expression modules with genome-wide PPI networks in pikeperch. Overall, we found four tissue-specific PPI networks (TSN) associated with 4 different tissues, including skin, liver, muscle and heart (Figure 6). These TSN involve between tree genes (skin, heart) and 13 genes (muscle). GO enrichment analysis identified no significantly enriched biological process and cellular component in these functional modules. However, GO terms associated with these genes mostly described biological processes specific to these tissues.

### 3.4. Positive Selection Analysis

**Figure 7:**
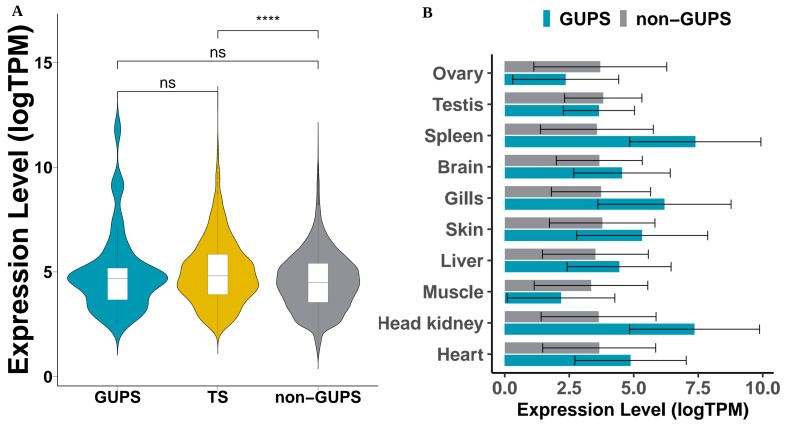
Expression levels of GUPS in pikeperch. (**A**), Violin plot comparing log transformed expression levels of GUPS, TS (Tissue-specific) and non-GUPS (all genes not under positive selection). The expression levels between GUPS and non-GUPS are not significantly different (Kruskal–Wallis-Test). (**B**), Log transformed mean expression of GUPS and non-GUPS in each tissue type. Statistical significance ns: Not significant; ****: Extremely significant (p<0.0001).

Six representative percid species were analysed for candidate GUPS (see Methods). Overall, we detected 43, 63, 137, 154, 152, and 124 putative candidate genes under selection pressure in *S. lucioperca, S. vitreus*, *P. flavescens, P. fluviatilis*, *E. spectabile*, and *E. cragini*, respectively (Table 5). Only two tissue-specific genes, *SLC13A2* and *VWA1* were found to be under positive selection. Although the expression levels of GUPS in *Sander lucioperca* did not significantly vary across tissues (One-way ANOVA, F<1), they were markedly expressed in higher levels in some tissues, such as head kidney, spleen, and gills (Figure 7B). Relative to tissue-specific genes (TS), GUPS were less tissue-specific (Kruskal–Wallis-Test, *p*-value < 0.0001). Although GUPS showed a higher tissue specificity index than genes not under positive selection (non-GUPS) (Kruskal–Wallis-Test, *p*-value < 0.01), and their expression levels were not significantly different (Figure 7A).

**Figure 8:**
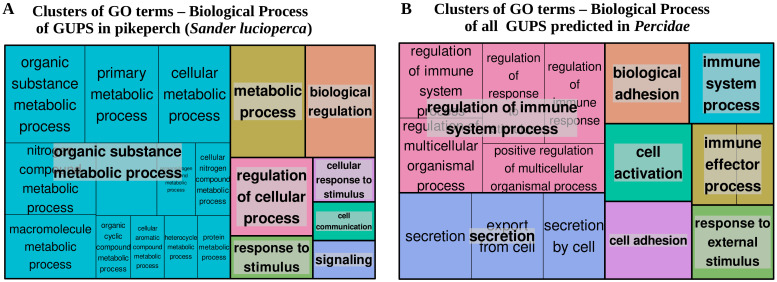
TreeMaps depicting GO terms (Biological Process) clusters GUPS in pikeperch (**A**) and in Percidae (all analysed species) (**B**), respectively. Each rectangle represents a cluster of related GO terms. The sizes of rectangle reflect the significance of the cluster (# of GO terms included in the cluster). Closely related GO terms are grouped together in a super-cluster of the same colour.

GO enrichment analysis of GUPS in *S.lucioperca*, *S. vitreus*, and *E. spectabile* revealed no significantly overrepresentated terms. Though, several GUPS in *Sander lucioperca* were associated with metabolic process, regulation of cellular process and response to stimulus. On the other hand, GUPS in *P. fluviatilis, P. flavescens* and *E. cragini* were significantly (FDR < 0.05) enriched with immune-related biological processes, including regulation of immune system process (GO:0002682), regulation of defense response (GO:0031347), myeloid leukocyte activation (GO:0002274), neutrophil degranulation (GO:0043312), leukocyte mediated immunity (GO:0002274) or neutrophil activation (GO:0042119) (Supplementary file S3). A broader overview of the functional terms associated with GUPS is shown on the treeMap in Figure 8, representing clusters of GO terms based on their context similarity. Each rectangle in the treeMap represents a cluster of GO terms associated with genes under positive selection. The size of rectangles reflects the significance of the of the cluster (i.e., the number of GO terms in the cluster). Closely related GO terms are clustered together in a supercluster of the same colour (Figure 8).

## 4. Discussion

Pikeperch is an emerging inland aquaculture species in Europe. For successful positioning this species in the European aquaculture industry, genomics insights can be harnessed in all stages of its domestication to understand its adaption biology, optimise breeding programs and improve commercial traits [52]. Hence, the comprehensive transcriptomics data presented here provide a key molecular resource for in-depth informing on developmental, evolutionary, and behavioural questions throughout the domestication process of the pikeperch.

The quality assessment of this new pikeperch transcriptome using BUSCO and various metrics suggest that a wide range of full length transcripts were resolved, since nearly 95% of single-copy orthologs in ray-finned fish (Actinopterygii) were covered by our assembly. Moreover, the merged assembly (EvidentialGenes) displayed the best contiguity and mappability metrics compared to the other assemblies generated with Trinity or rnaSPAdes assemblers (Figure 1). It has been demonstrated in similar studies that combining transcriptome assemblies from multiple assemblers or assembly approaches yields significantly better and optimised results compared to assemblies built with a single assembler [28,53,54].

Tissue-specific gene expression is a well-known biological phenomenon by which the genome expresses differentiated transcriptomes among tissues and cell types. Therefore, tissue-specific protein-coding transcripts can explain the difference in the composition and complexity of the transcriptomes of different tissues, as well as provide clues to detecting key pathways and physiological and regulatory processes unique in a tissue [17]. Our analysis using the tissue specificity index (τ) with RNA-Seq expression profiles of tissues from 10 vital pikeperch organs allowed us to establish the first catalog of tissue-specific genes and to capture their specific metabolic process. In our dataset, testis and brain tissues had the most complex transcriptomes, while ovary and testis tissues featured the highest number of tissue-specific protein-coding genes, accounting for 19% and 13% of all tissue-specific genes, respectively (Figure 4E). This trend is comparable with previous studies across different taxa including domesticated animals. We denote studies in pigs [55], salmons [19,20], and crucian carp [18], or well studied models such as rats [56] and mice [57]—birds [17] and even on higher-order mammals such as humans [43,58,59,60], where brain and gonads tissues consistently expressed the most tissue-specific transcripts. These suggest a conserved tissues-specific expression pattern across main vertebrate taxa and lineages.

Among tissue-specific genes, we identified 151 transcription factors (TFs) validated in diverse fish species, 15 immune-related genes (IRG), 3 hypoxia-related genes (HRG), and only 2 GUPS, suggesting that these important classes of genes are less likely to be uniquely expressed in a specific tissue. In contrast, these genes were similarly expressed in all tissues (Expressed-In-All), or moderately expressed in a subset of tissues (Mixed) (Figure 2). More than one third (35%) of detected TFs, IRG, HRG, and GUPS were either classified as “Expressed-In-All” or “Mixed”, while only 9% were “Tissue-Specific”. However, this is an expected observation, in that transcription factors, for example, are more likely to be ubiquitously expressed, as they are regulatory proteins acting as housekeeping genes in different tissue types. In addition, TFs identified as tissue-specific in our data trigger the expression of genes involved in highly specialized organ-limited functions. For instance, the GATA transcription factors family including *GATA4*, *GAT5*, and *GATA6*, which are known to play a key role in cardiac development and cardiomyocyte gene expression, were specifically expressed in the heart tissues of pikeperch. Similarly, SOX32 (SRY-box transcription factor 32) and HSF5 (Heat Shock Factor 5), which are known TF playing an essential role in spermatogenesis in Zebrafish [61] and other fish species [62], were testis-specific in pikeperch. Lastly, we want to highlight three hypoxia-related genes including the hypoxia-inducible factor prolyl hydroxylase 2 (EGLN1), ceruloplasmin (CP), and solute carrier family 2 (SLC2A2), which have been identified as tissue-specific (in heart and liver, respectively). These transcription factors are known to regulate the expression of hypoxia-responsive genes [63,64,65].

Functional analysis revealed that tissue-specific genes were largely associated with important biological process and pathways involved with the corresponding tissue-specific physiological functions (Figure 3). For example, ovary-specific genes were largely associated with oocyte meiosis and cell cycle process. Genes specifically expressed in the spleen and head kidney were mostly involved in immune-related biological processes. Another notable example is heart and skeletal muscle specific genes, which were largely associated with muscle contraction and heart development, respectively (Figure 3). This functional characterization is also expected and is in line with previous findings in comparative transcriptome analysis between tissues [17,60]. More interestingly, we observed high similarities and correlations of expression patterns between some tissues, reflecting biological identity, coordinated processes, or functional convergence between these tissues. All tissues clustered together share either functional, morphological, physiological, or developmental features. For example, the nearly perfect overlap between gills and skin specific genes as revealed by UMAP clustering is probably explained by the facts that gills as well as skin are enriched with epithelial cells and both potentially act as respiratory organs of teleosts. The high correlation (Pearson’s R > 0.8) between heart, muscle, and liver-specific genes is probably due to coordinated metabolic activities in these tissues, such as lipogenis in the liver and fatty acid oxidation in skeletal muscle cells [66]. Similarly, the testis and ovary, which are reproductive system organs, showed strongly correlated expression patterns. The observed correlation between tissues is corroborated by the genome-wide tissue-specific co-expression analysis. Namely, the co-expression module M3 is specific to the testis and ovary, while the module M5 is specific to the head kidney and spleen tissues (Supplementary file S4). Collectively, these results do not only suggest a high reliability and repeatability of our RNA-Seq analysis, but also endorse the correct classification of genes expressed in a tissue-specific manner.

Tissue-specific genes are a good starting point to quantify how tissues and organs differ in their expression profiles and interrogate how gene expression shape the phenotype and function of various tissues in an organism. However, a strong limitation of functional inference of tissue-specific genes is that each gene is analysed independently, while in reality, no gene is able to perform biological function on its own. Most genes tend to operate within functional modules and complex networks of interacting proteins. Tissue-specific gene expression can help us to predict tissue-specific interaction networks and infer their unique functions more precisely. Moreover, functional modules inferred from tissue-specific networks are more specific than those inferred from global PPI networks. In order to discover co-expressed network modules that are significantly associated with different pikeperch tissues, we constructed tissue-specific PPI networks based on differentially co-expressed genes, that is, co-expression modules induced by tissue-specific genes. As shown in the results section, only 4 tissues (heart, muscle, liver, and skin) significantly displayed tissue-specific PPI networks. The functional pathways and biological processes associated with tissue-specific functional networks module were similar to the aforementioned functions of tissue-specific genes. In particular, hub genes obtained from the tissue-specific PPI network analyses were closely involved in biological functions typical to the specific tissues. For example, the protein *TNNT2A* (cardiac muscle troponin T type 2a), a top hub gene in the muscle-specific PPI network, is predicted to be a structural constituent of the cytoskeleton. Moreover, *TNNT2A* is the tropomyosin-binding subunit of the troponin complex located on the thin filament of striated muscle and regulates muscle contraction [67,68]. Besides, the secreted protein FGA (fibrinogen alpha chain), the main hub gene in the liver-specific PPI network, is known to play a key role in teleost haemostasis [69,70]. Overall, Gene Ontology and pathways annotation of the functional network modules revealed functionality coherent with tissue-specific processes.

In an attempt to associate tissue specificity with rapidly evolving genes in pikeperch, we predicted 43 GUPS in the pikeperch-specific lineage, compared to 5 other percid species (see Methods). While no significantly overrepresented biological process was found among the putative positively selected genes in pikeperch, GO terms associated with these genes mostly comprise metabolic processes and cellular response to stimuli (Figure 8). Subsequent functional analyses would broadly characterize their adaption features in the life history of pikeperch. Unexpectedly, only two GUPS were tissue-specific (liver-specific) and no significant correlation between positive selection and tissue-specificity was established. Moreover, most of the detected GUPS (27/32) were classified as “Mixed” (19) or “Expressed-In-All” (8), and tended to be expressed at lower levels relative to tissue-specific genes (Figure 7A). This result strengthens the hypothesis that genes under natural selection are more likely to be expressed at moderate or lower levels [71,72]. On the other hand, highly tissue-specific genes are significantly expressed at higher levels. Thus, we can hypothesise that high tissue-specificity in *Percidae* might release some genes from selection pressure.

Transcriptomic resources in aquaculture provide a solid basis to develop tissue-specific tools for, e.g., diagnosis and the recording of stress parameters. They also empower proteomics analysis to validate the functions of genes underlying key production traits. For example, subsequent analyses could leverage tissue-specific expression and co-expression patterns to explore markers in any tissue that may play a central role in the timing of breeding. Additionally, multitissue expression studies are useful for investigating feed efficiency and growth-related genes. All in all, this transcriptomics study will be useful in improving our understanding of the adaptation, development, growth and metabolism parameters of pikeperch, which can inform aquaculturists in making customized adjustments to environmental conditions, such as hypoxia, temperature, nutrition, and salinity.

## 5. Conclusions

In the present study, we first reported a multitissue high-quality reference transcriptome from 10 pikeperch vital tissues along with a comprehensive landscape of tissue specific expression and co-expression networks for classifying protein-coding genes regarding to their unique expression pattern across tissues. We then characterized the specific tissue function by identifying functional pathways and biological processes associated with tissue-specific genes and network modules. Finally, we shed light into the genetic evolutionary history of pikeperch by predicting putative genes under selection pressure, including known hypoxia-related genes, immune-related genes, and transcription factors. Collectively, the transcriptomics resources presented in this study can be useful for understanding the mechanisms of organ biology and the roles of specific metabolic cycles in different tissues. This knowledge will then lay a framework for investigating important production and domestication traits in the pikeperch. Ultimately, the transcriptome dataset will complement the amount of information of this aquaculture species in public data repositories.

## Figures and Tables

**Table 1:** Summary statistics of paired-end RNA-Seq reads yielded from 18 libraries of ten different pikeperch tissues using Illumina NovaSeq 6000 System.

Libraries	No. of Raw Reads	Q30 Raw Reads (%)	No. of Clean Reads	Q30 Clean Reads (%)
Heart-1	33,908,652	93.98	30,984,679	97.42
Heart-2	35,031,697	94.16	33,180,437	97.49
Head kidney-1	33,002,587	93.95	30,015,644	97.51
Head kidney-2	39,938,681	94.16	36,623,609	97.50
Muscle-1	35,047,416	94.74	32,410,797	97.52
Muscle-2	39,426,896	94.32	36,506,458	97.53
Liver-1	32,566,471	94.20	30,209,964	97.42
Liver-2	35,071,990	94.11	32,007,540	97.65
Brain-1	40,422,234	93.58	37,106,005	97.40
Brain-2	35,567,608	93.46	36,458,085	97.41
Skin-1	37,989,173	94.21	35,172,646	97.42
Skin-2	40,633,032	94.34	37,687,356	97.47
Gills-1	38,586,131	93.97	35,630,505	97.42
Gills-2	39,427,046	94.51	36,458,085	97.41
Spleen-1	45,127,155	94.00	41,790,579	97.36
Spleen-2	33,329,198	94.27	30,504,153	97.44
Ovary	37,553,020	94.38	34,742,133	97.51
Testis	48,694,199	93.90	44,903,971	97.37
**Average**	**37,851,288**	94.12	**35,132,924**	97.45
**Total**	**681,323,186**	**—**	**632,392,646**	**—**

**Table 2:** Summary of pikeperch transcriptome assembly and assessment.

	Trinity	rnaSPAdes	Hisat2 + StringTie2	EvidentialGene
Number of contigs	438,462	295,387	79,936	**56,302**
Cumulative contigs length (Mb)	399.28	502.46	299.28	**85.73**
Mean contigs length (bp)	910.65	1701.05	**3744.49**	1522.81
N50 contigs length (bp)	1340	3436	**4934**	1977
Largest contig (bp)	70,079	**80,089**	78,909	79,815
∑ contigs > 1 Kb (%)	57.11	83.16	**98.31**	80.51
% of FL transcripts	60.57	72.84	**89.52**	86.73
% of transcripts with ORFs	76.73	80.53	**88.84**	85.07
% of BUSCO complete	80.27	96.58	96.62	**96.87**
% of transcripts with NCBI NR hits	72.83	78.04	86.27	**88.35**
% of transcripts with Swiss-Prot hits	55.76	60.23	75.86	**78.57**
Mapping rate RNA-Seq reads (%)	83.92	84.75	**90.86**	88.15

**Table 3:** Classification of protein-coding genes based on transcript expression levels and their index of tissue specificity (τ) across 10 pikeperch tissues.

Category	No. of Genes	Fraction of Detected Genes (%)
Tissue-Specific	2930	15.00
Group-Enriched	3809	19.50
Expressed-in-All	5810	29.80
Mixed	6970	35.70
Total detected	19,541	100

**Table 4:** Tissue-specific (differential) co-expression modules with their hub genes.

Module	No. Genes	Tisssue-Specific Upregulation	Hubs (Gene Symbol)
M1	55	Liver	*C3, AFP4, C1QTNF3*
M2	53	Muscle	*PYGM, TNNT3A, TRIM21, MYLPFA*
M3	27	Ovary, Testis	*SERPINA12, ALOX12B, LOC116046623*
M4	21	Skin	*RPS7, RPS3A, RPL5, RPL13A, RPL7A*
M5	19	Head kidney, Spleen	*HBZ, NPRL3, AQP8A, HBB2*
M6	15	Gills, Skin	*LOC116046623, ZG16B, MPO*
M7	14	Heart	*TNNT2A, MYBPC3, TNNC1A, TNNI1, TPM4A*

**Table 5:** Statistics on lineage-specific positive selection in the six representative Percidae species.

Branch	No of. CDS	No. of GUPS	Mean ω ($d_{N}/d_{S}$)	Avg No. of Sites
*Sander lucioperca*	56,899	43	5.11	6.63
*Sander vitreus*	34,187	63	4.08	9.16
*Perca flavescens*	43,150	137	3.41	8.41
*Perca fluviatilis*	50,212	154	5.97	7.80
*Etheostoma spectabile*	45,699	152	4.07	9.10
*Etheostoma cragini*	45,199	124	3.24	9.22

## Data Availability

Raw RNA-Seq reads are openly available at NCBI SRA (BioProject PRJNA752979). The transcriptome shotgun assembly has been deposited at DDBJ/EMBL/GenBank under the accession GJIW00000000. The version described in this paper is the first version, GJIW01000000. Codes used for data analysis as well as generated figures, tables, and extended methods are available on github (https://github.com/bbalog87/Pikeperch_transcriptomics, accessed on 28 August 2021).

## Supplementary Materials

The following are available online at https://www.mdpi.com/article/10.3390/cells10092289/s1, Figure S1: balloon plot of KEGG pathways associated with tissue-specific genes in pikeperch. Supplementary file S1: RNA-Seq sample design. Supplementary file S2: Complete classification of pikeperch protein-coding genes. Supplementary file S3: Functional annotation of all GUPS in *Percidae*. Supplementary file S4: Differential co-expression analysis—PDF report.
