# Supplementary material for: Comprehensive Characterization of Multitissue Expression Landscape, Co-Expression Networks and Positive Selection in Pikeperch
Source: Cells. 2021 Sep 2;10(9):2289. doi: 10.3390/cells10092289 (PMC8471114; doi:10.3390/cells10092289)
Supplement: Supplementary file 1 [file cells-10-02289-s001.zip › Supplementary File S4.pdf]

CEMiTool

Contents

1 Differential Co-expression analysis in Pikeperch **1**
1.1 Modules 1
1.2 Profile Plot 1
1.3 Gene Set Enrichment Analysis 6
1.4 Over Representation Analysis 7
1.5 Interaction Network 13
1.6 Parameters 15

1 Differential Co-expression analysis in Pikeperch

1.1 Modules

```
## PhantomJS not found. You can install it with webshot::install_phantomjs(). If it is installed, please
```

1.2 Profile Plot

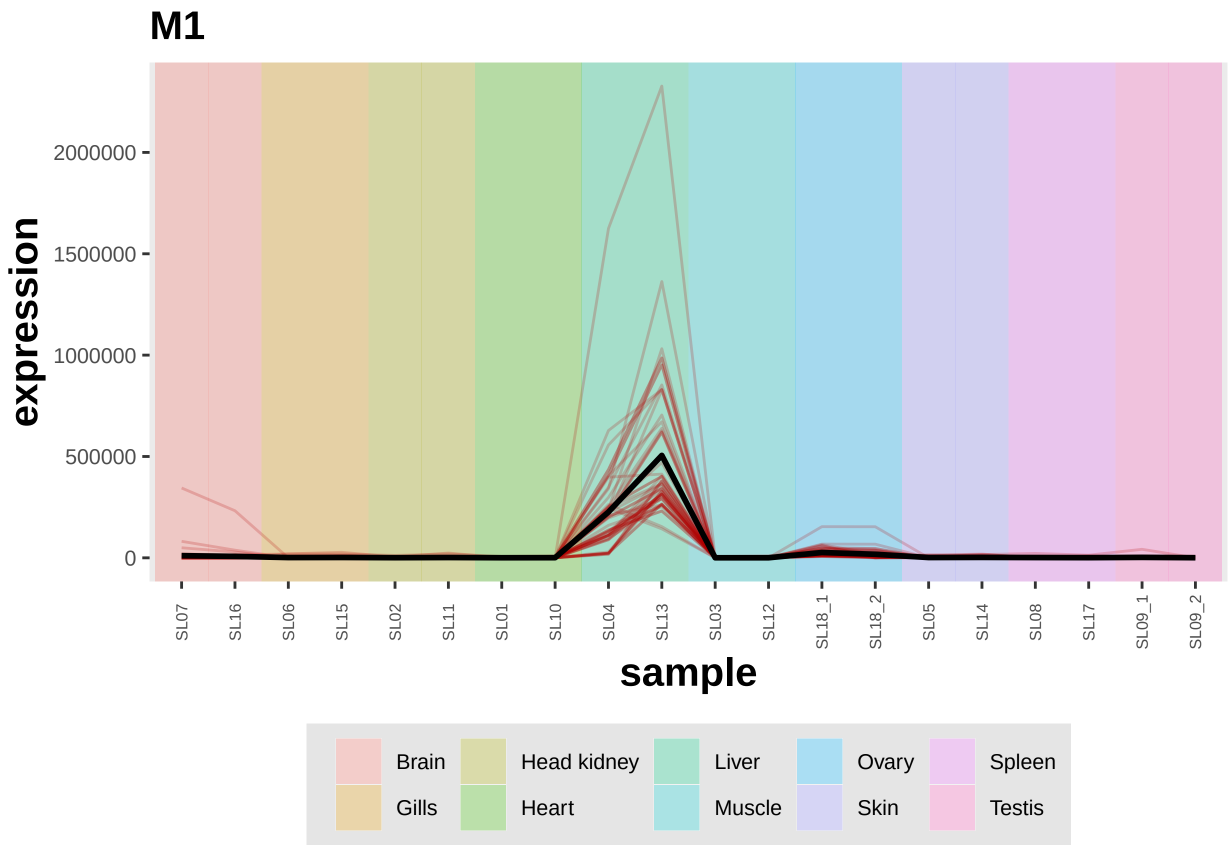

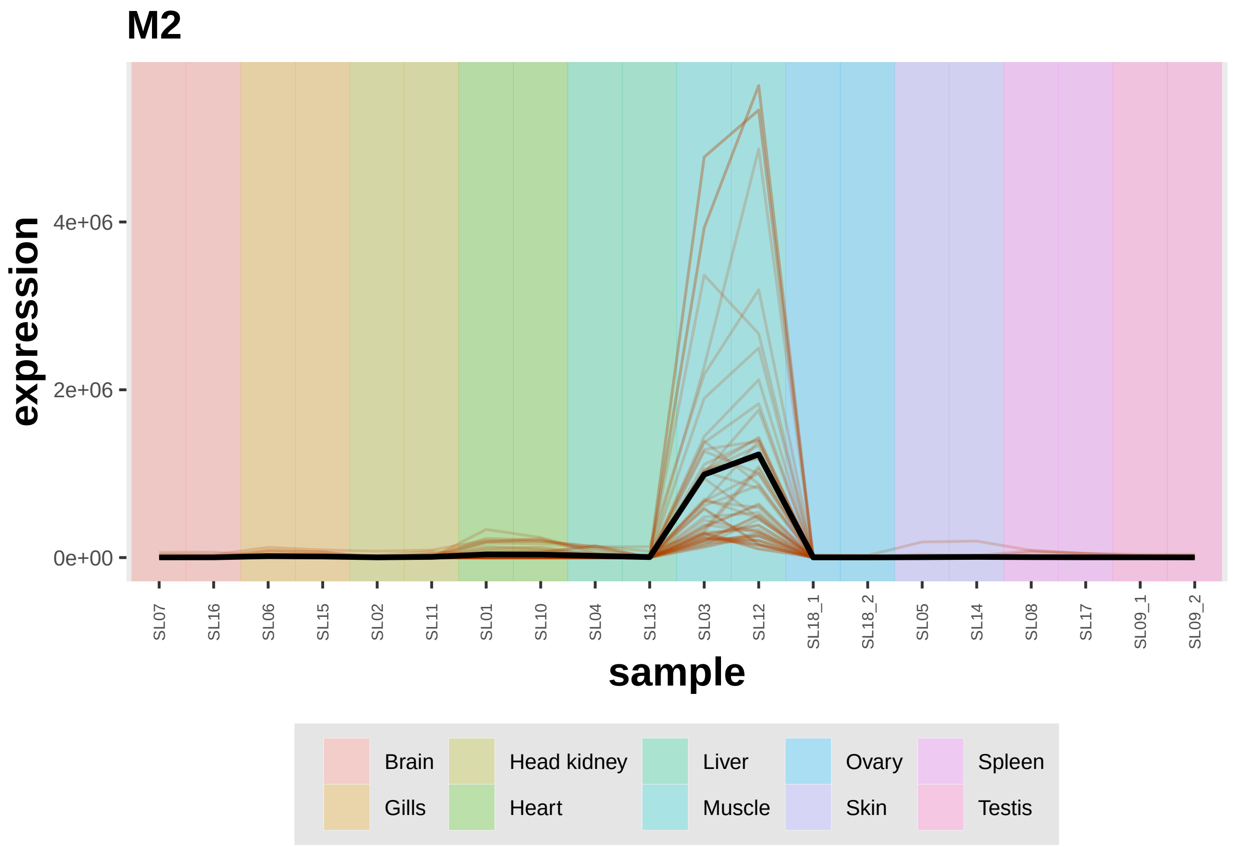
M2
expression
4e+06
2e+06
0e+00
SL07
SL16
SL06
SL15
SL02
SL11
SL01
SL10
SL04
SL13
SL03
SL12
SL18_1
SL18_2
SL05
SL14
SL08
SL17
SL09_1
SL09_2
sample
Brain
Gills
Head kidney
Heart
Liver
Muscle
Ovary
Skin
Spleen
Testis

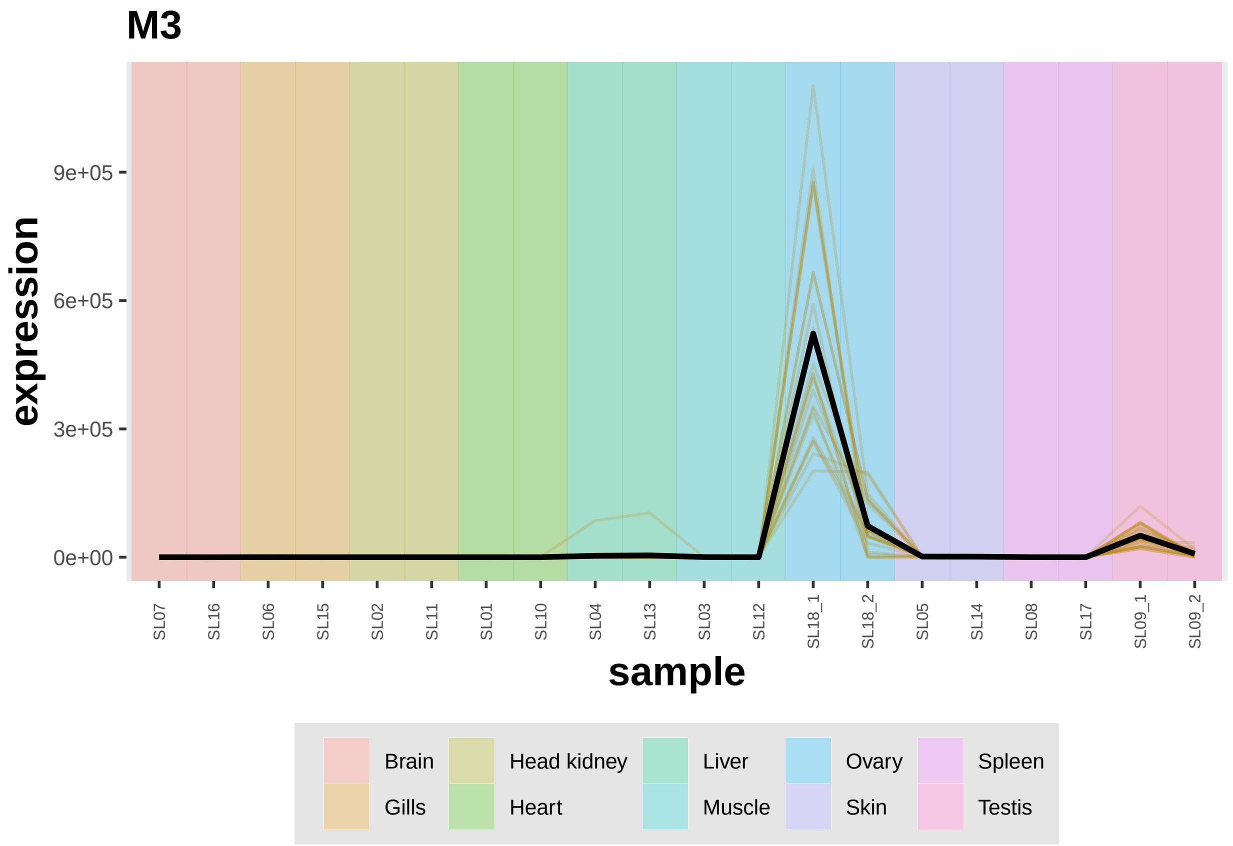
M3
expression
9e+05
6e+05
3e+05
0e+00
SL07
SL16
SL06
SL15
SL02
SL11
SL01
SL10
SL04
SL13
SL03
SL12
SL18_1
SL18_2
SL05
SL14
SL08
SL17
SL09_1
SL09_2
sample
Brain
Gills
Head kidney
Heart
Liver
Muscle
Ovary
Skin
Spleen
Testis

M4

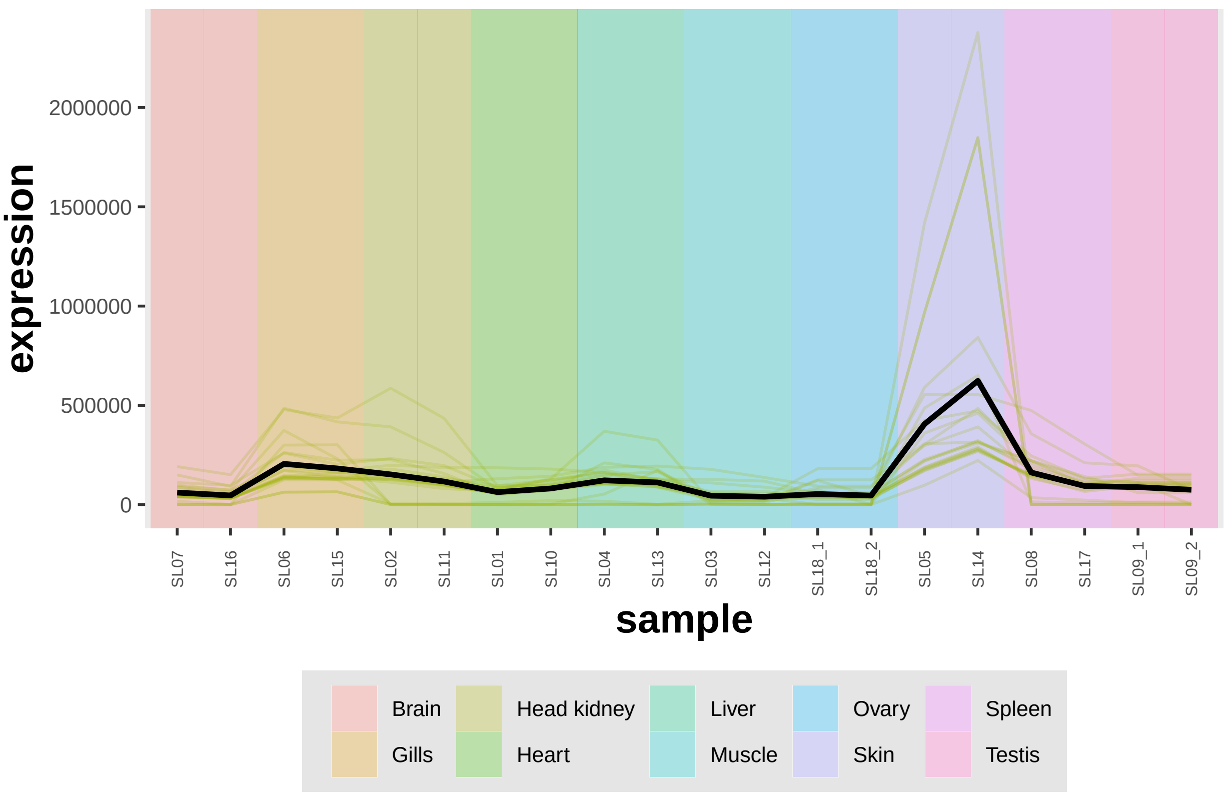
expression
2000000
1500000
1000000
500000
0
SL07
SL16
SL06
SL15
SL02
SL11
SL01
SL10
SL04
SL13
SL03
SL12
SL18_1
SL18_2
SL05
SL14
SL08
SL17
SL09_1
SL09_2
sample
Brain
Gills
Head kidney
Heart
Liver
Muscle
Ovary
Skin
Spleen
Testis

M5

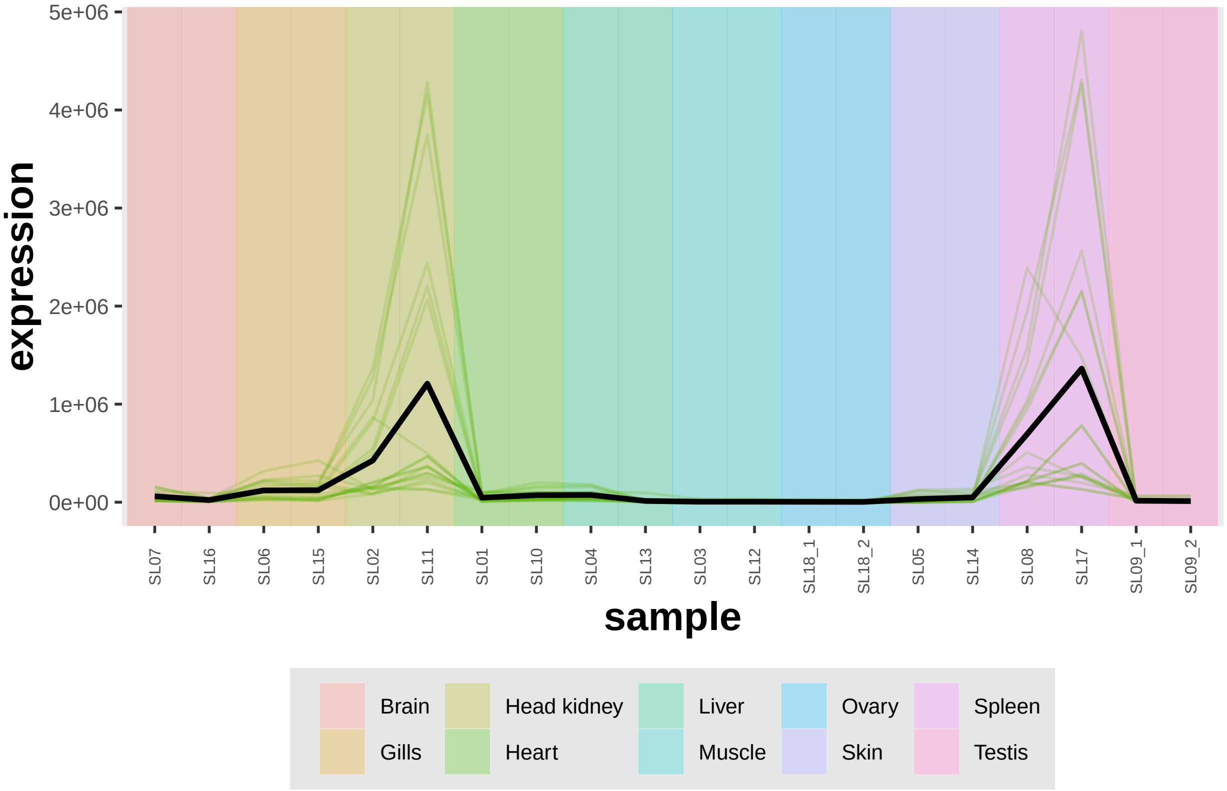
expression
5e+06
4e+06
3e+06
2e+06
1e+06
0e+00
SL07
SL16
SL06
SL15
SL02
SL11
SL01
SL10
SL04
SL13
SL03
SL12
SL18_1
SL18_2
SL05
SL14
SL08
SL17
SL09_1
SL09_2
sample
Brain
Gills
Head kidney
Heart
Liver
Muscle
Ovary
Skin
Spleen
Testis

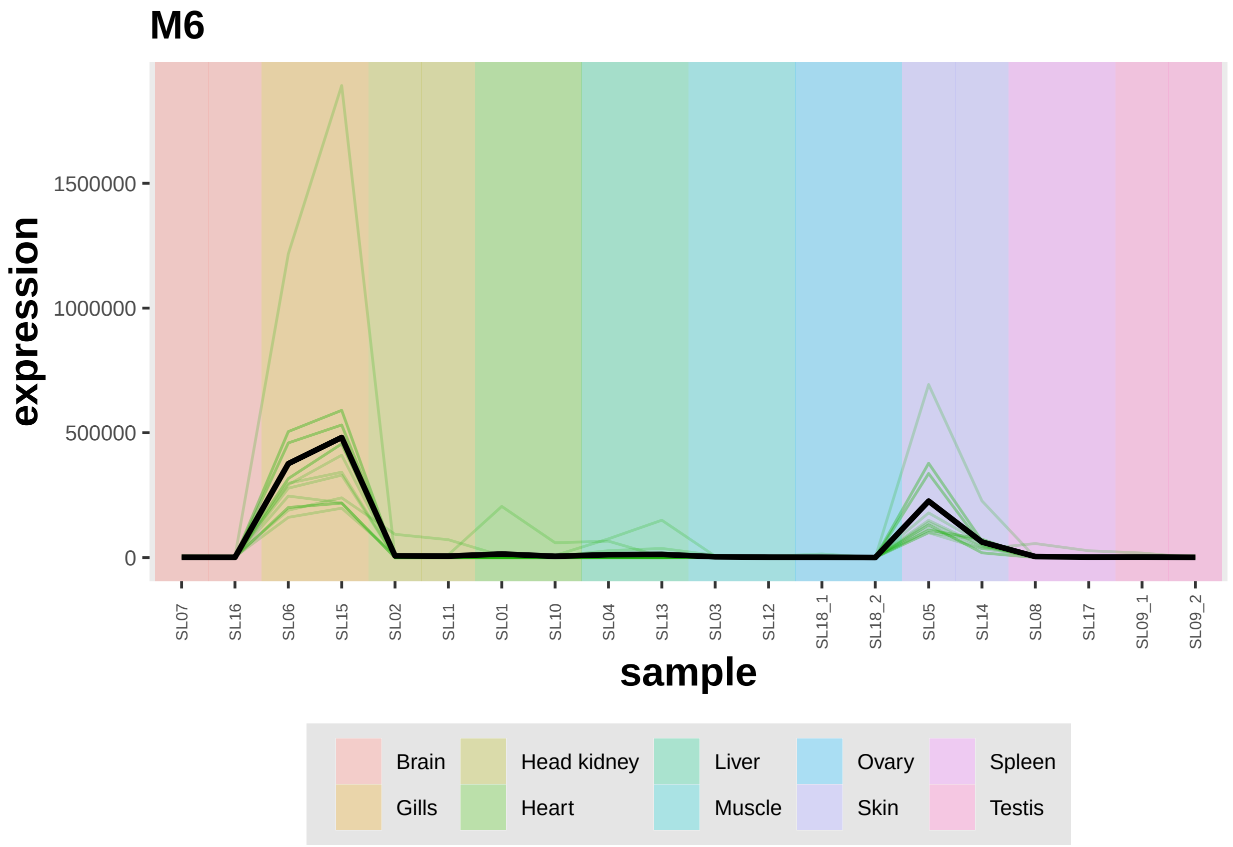
M6
expression
1500000
1000000
500000
0
SL07
SL16
SL06
SL15
SL02
SL11
SL01
SL10
SL04
SL13
SL03
SL12
SL18_1
SL18_2
SL05
SL14
SL08
SL17
SL09_1
SL09_2
sample
Brain
Gills
Head kidney
Heart
Liver
Muscle
Ovary
Skin
Spleen
Testis

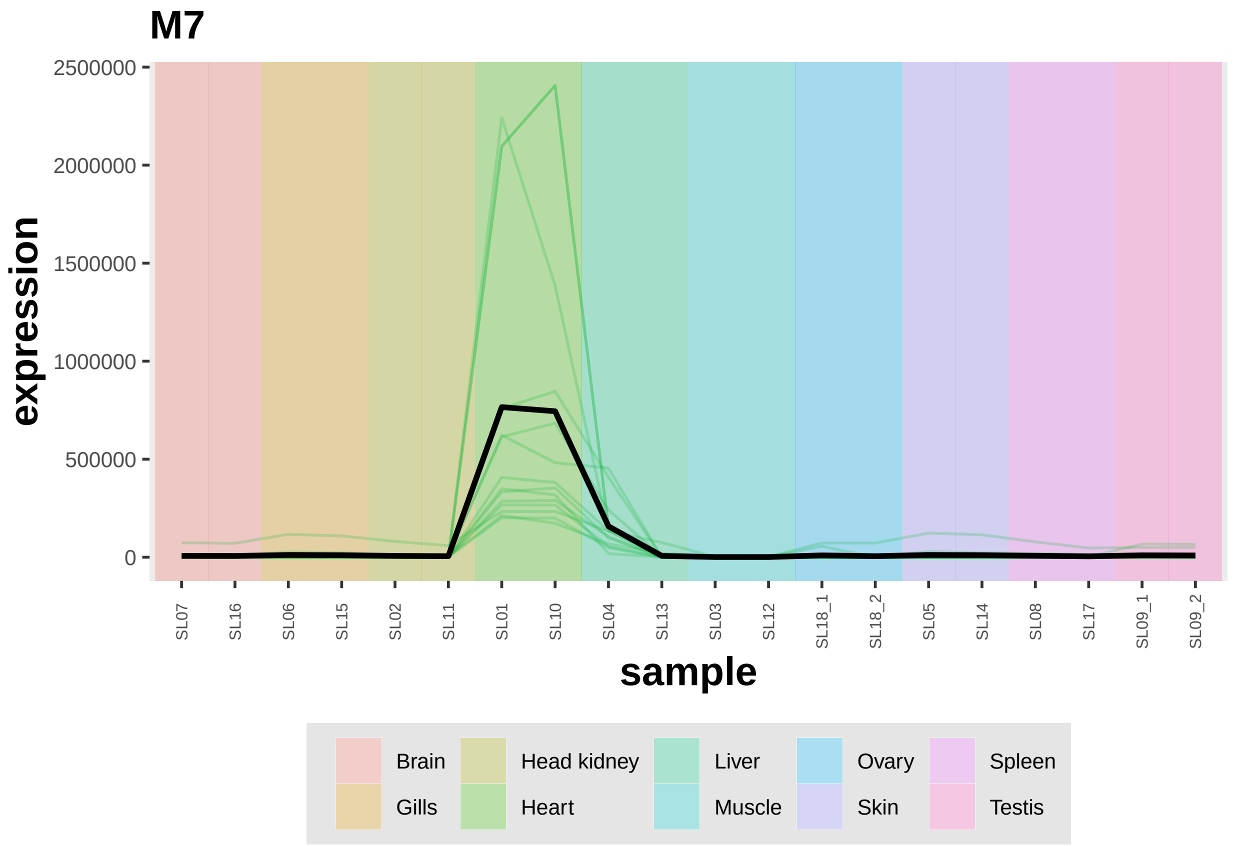
M7
expression
2500000
2000000
1500000
1000000
500000
0
SL07
SL16
SL06
SL15
SL02
SL11
SL01
SL10
SL04
SL13
SL03
SL12
SL18_1
SL18_2
SL05
SL14
SL08
SL17
SL09_1
SL09_2
sample
Brain
Gills
Head kidney
Heart
Liver
Muscle
Ovary
Skin
Spleen
Testis

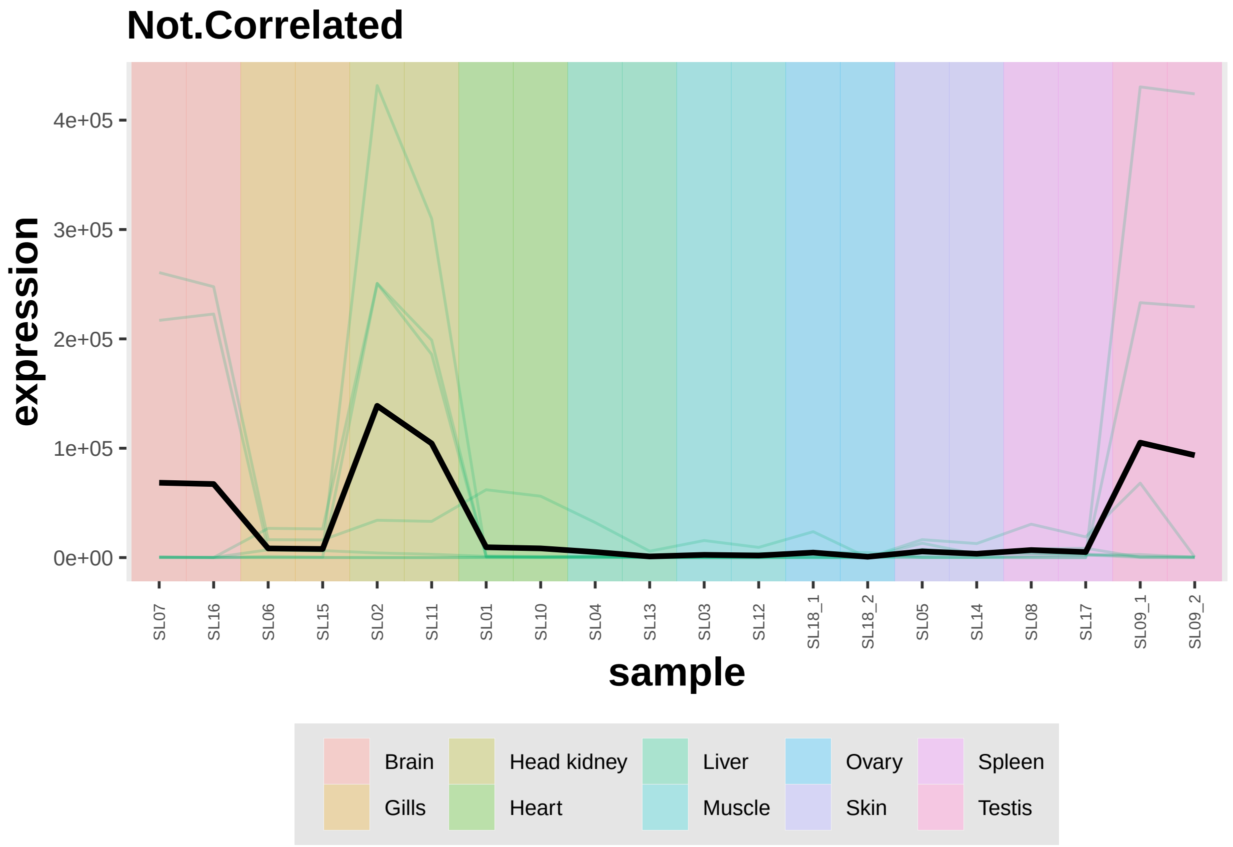
Not.Correlated
expression
4e+05
3e+05
2e+05
1e+05
0e+00
SL07
SL16
SL06
SL15
SL02
SL11
SL01
SL10
SL04
SL13
SL03
SL12
SL18_1
SL18_2
SL05
SL14
SL08
SL17
SL09_1
SL09_2
sample
Brain
Gills
Head kidney
Heart
Liver
Muscle
Ovary
Skin
Spleen
Testis

1.3 Gene Set Enrichment Analysis

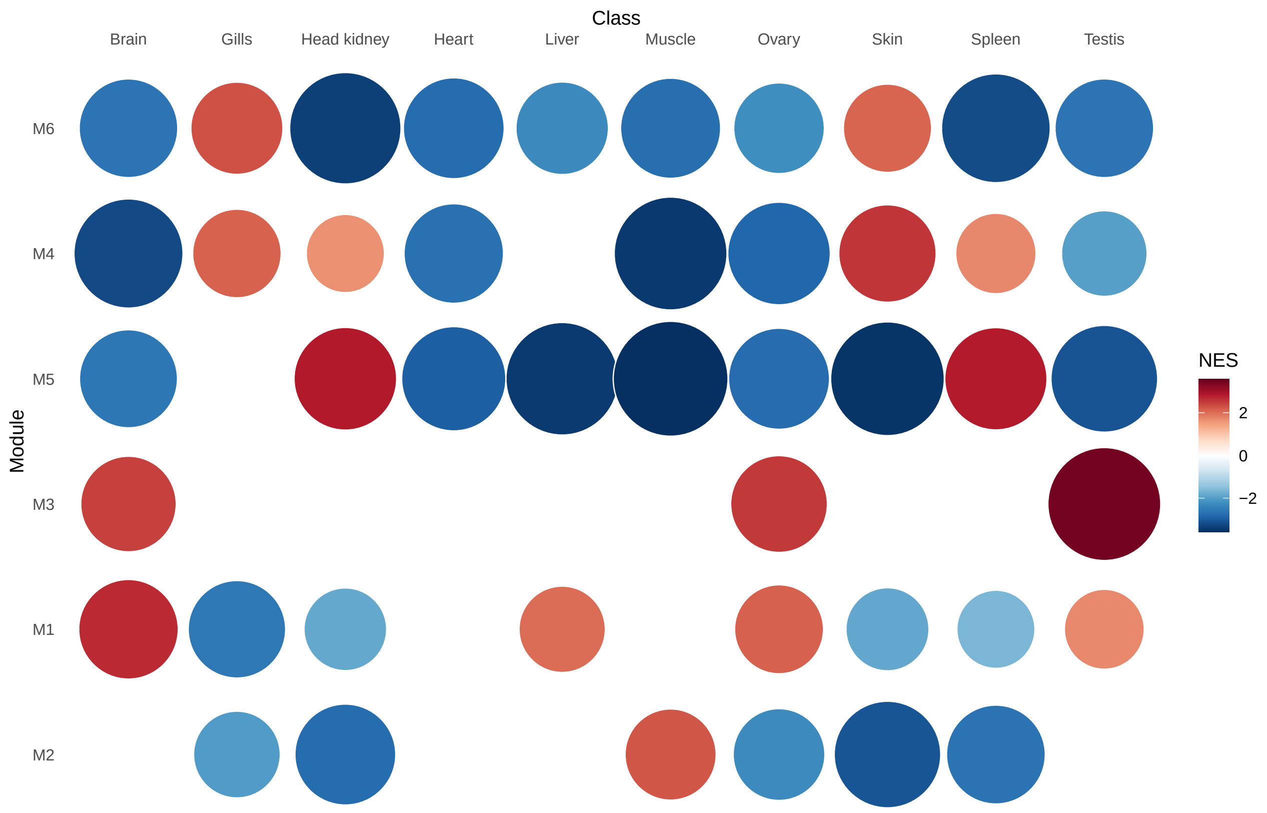

1.4 Over Representation Analysis

1.4.1 M1

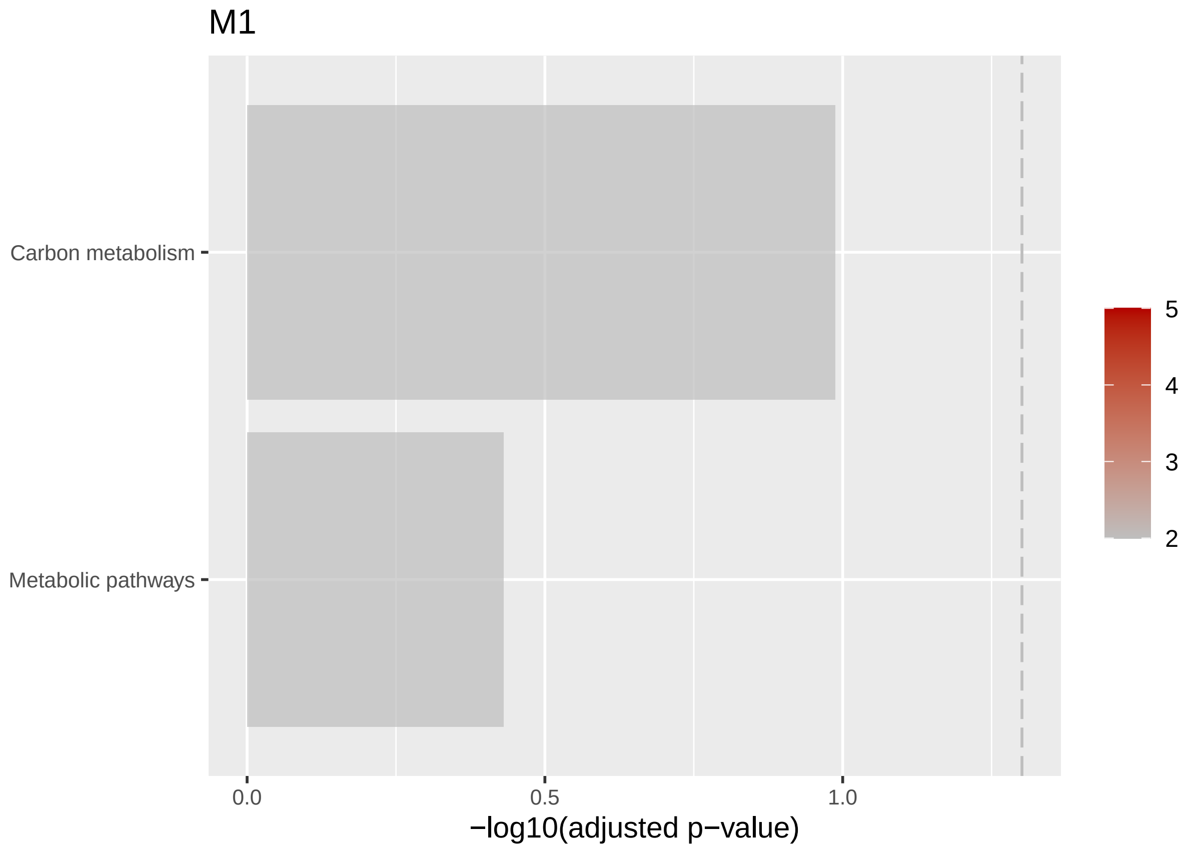

1.4.2 M2

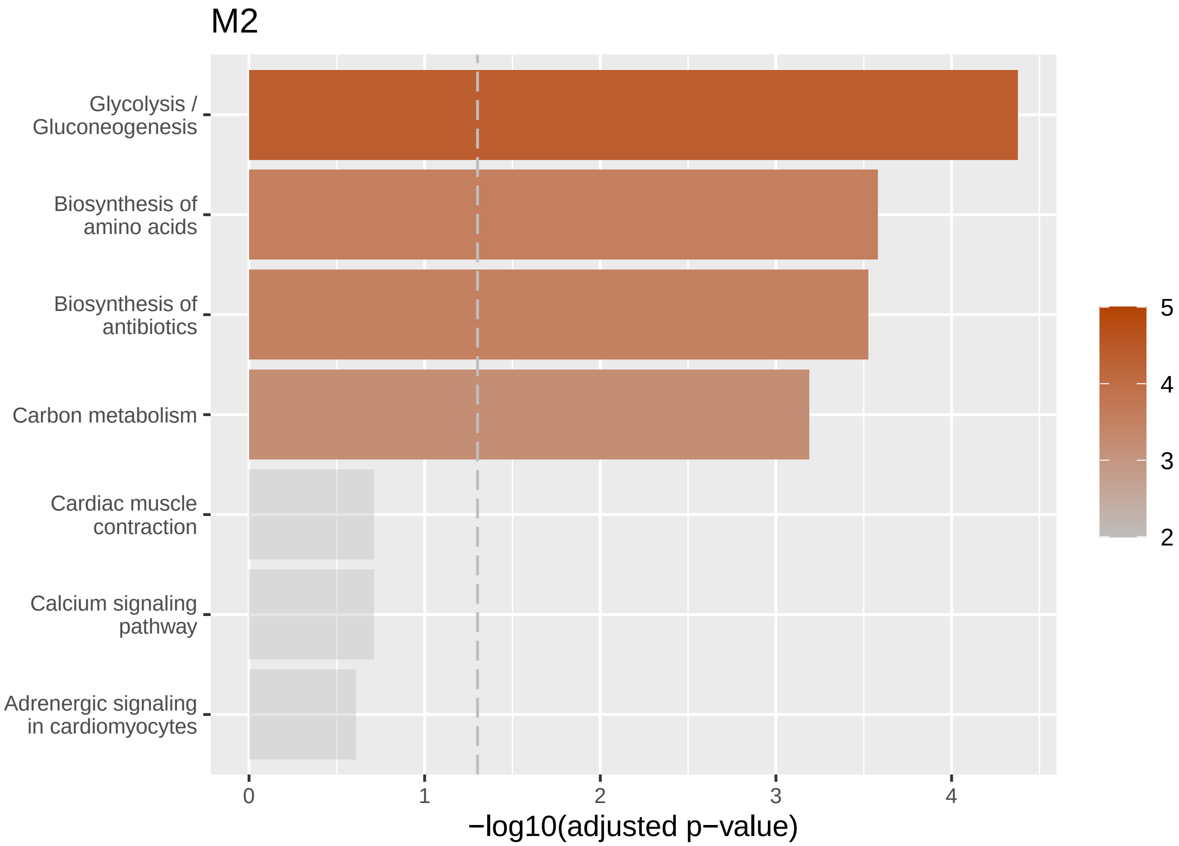
M2
Glycolysis / Gluconeogenesis
Biosynthesis of amino acids
Biosynthesis of antibiotics
Carbon metabolism
Cardiac muscle contraction
Calcium signaling pathway
Adrenergic signaling in cardiomyocytes
0
1
2
3
4
−log10(adjusted p−value)
5
4
3
2

1.4.3 M4

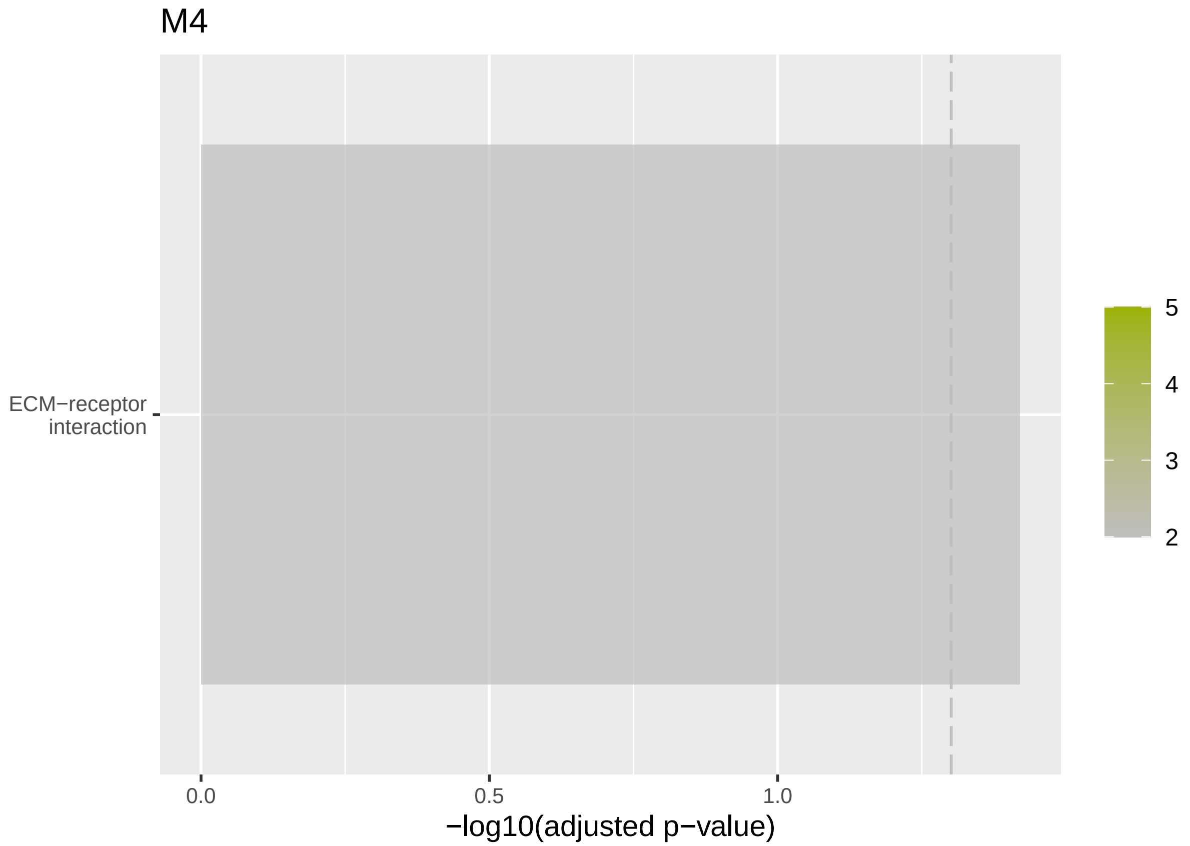

M4
ECM−receptor interaction
0.0
0.5
1.0
−log10(adjusted p−value)
5
4
3
2

1.4.4 M5

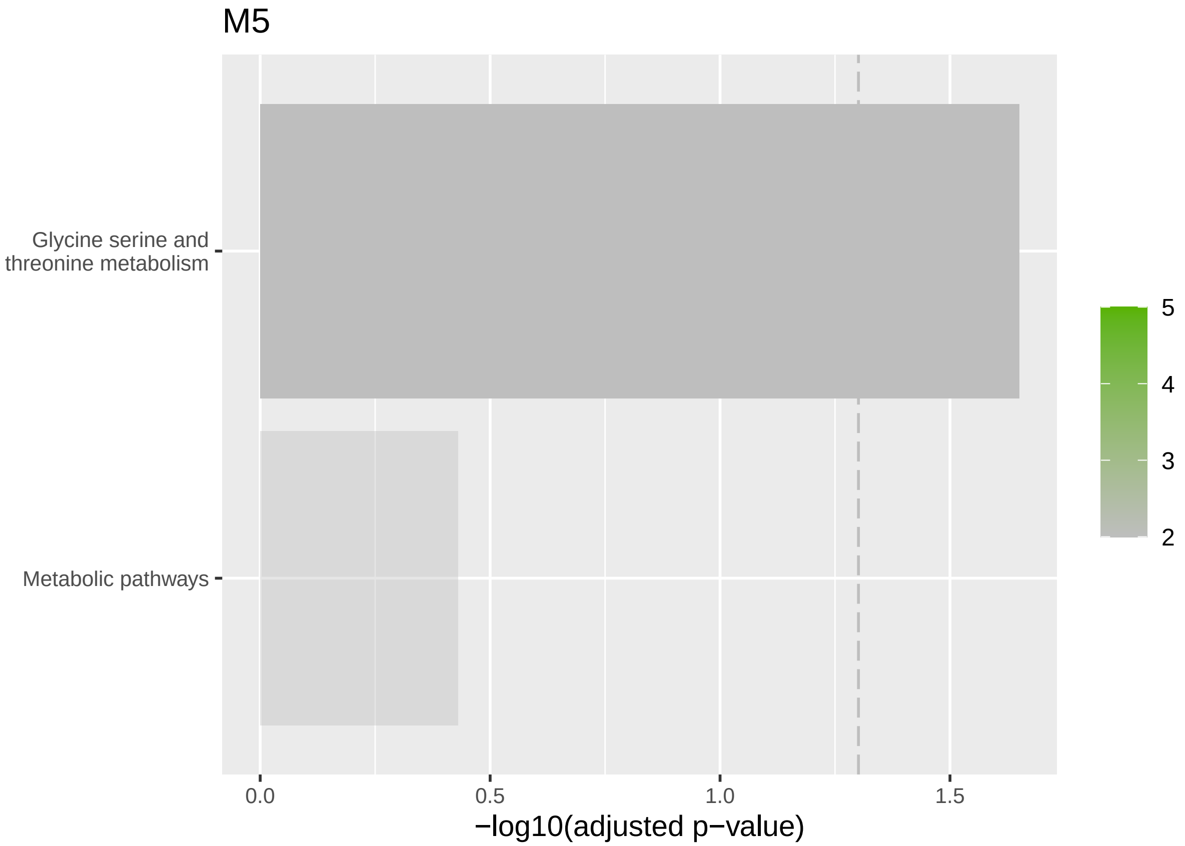
M5
Glycine serine and threonine metabolism
Metabolic pathways
0.0
0.5
1.0
1.5
−log10(adjusted p−value)
5
4
3
2

1.4.5 M6

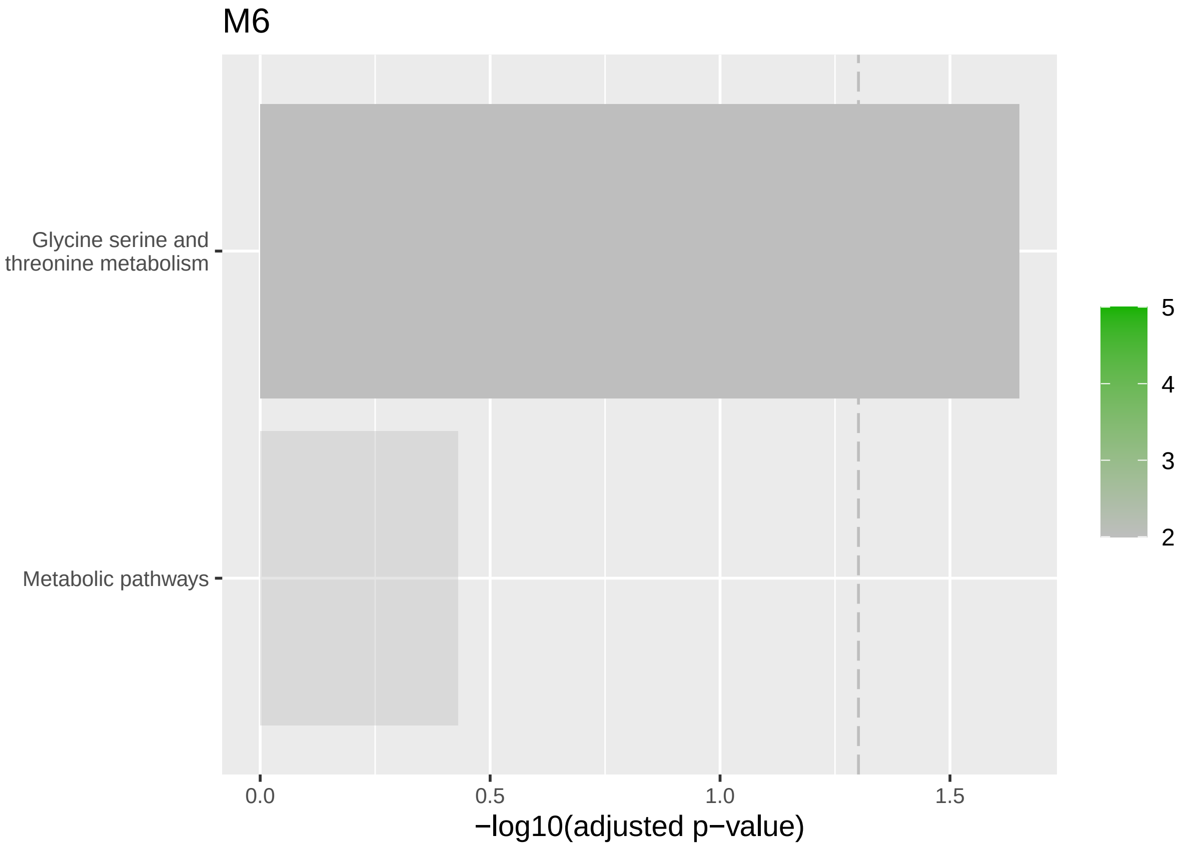
M6
Glycine serine and threonine metabolism
Metabolic pathways
0.0
0.5
1.0
1.5
−log10(adjusted p−value)
5
4
3
2

1.4.6 M7

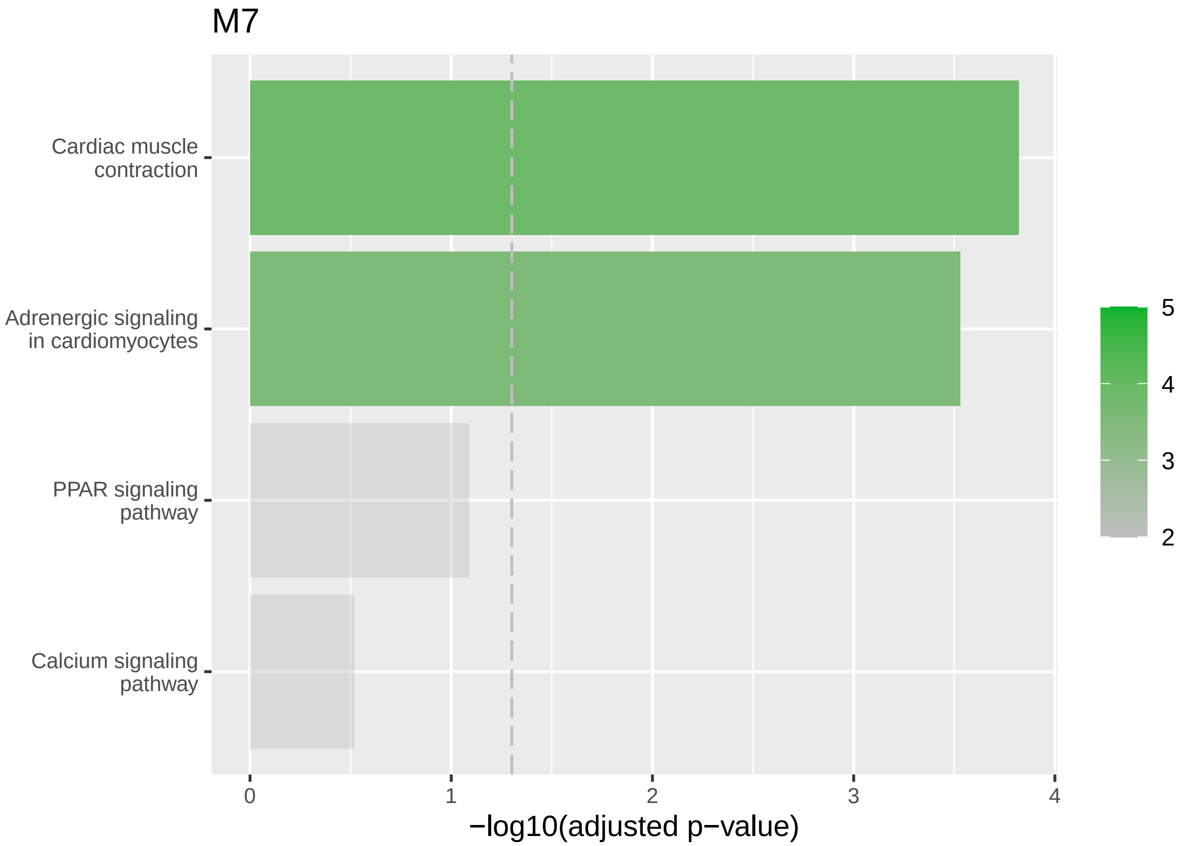

M7
Cardiac muscle contraction
Adrenergic signaling in cardiomyocytes
PPAR signaling pathway
Calcium signaling pathway
0
1
2
3
4
−log10(adjusted p−value)
5
4
3
2

1.5 Interaction Network

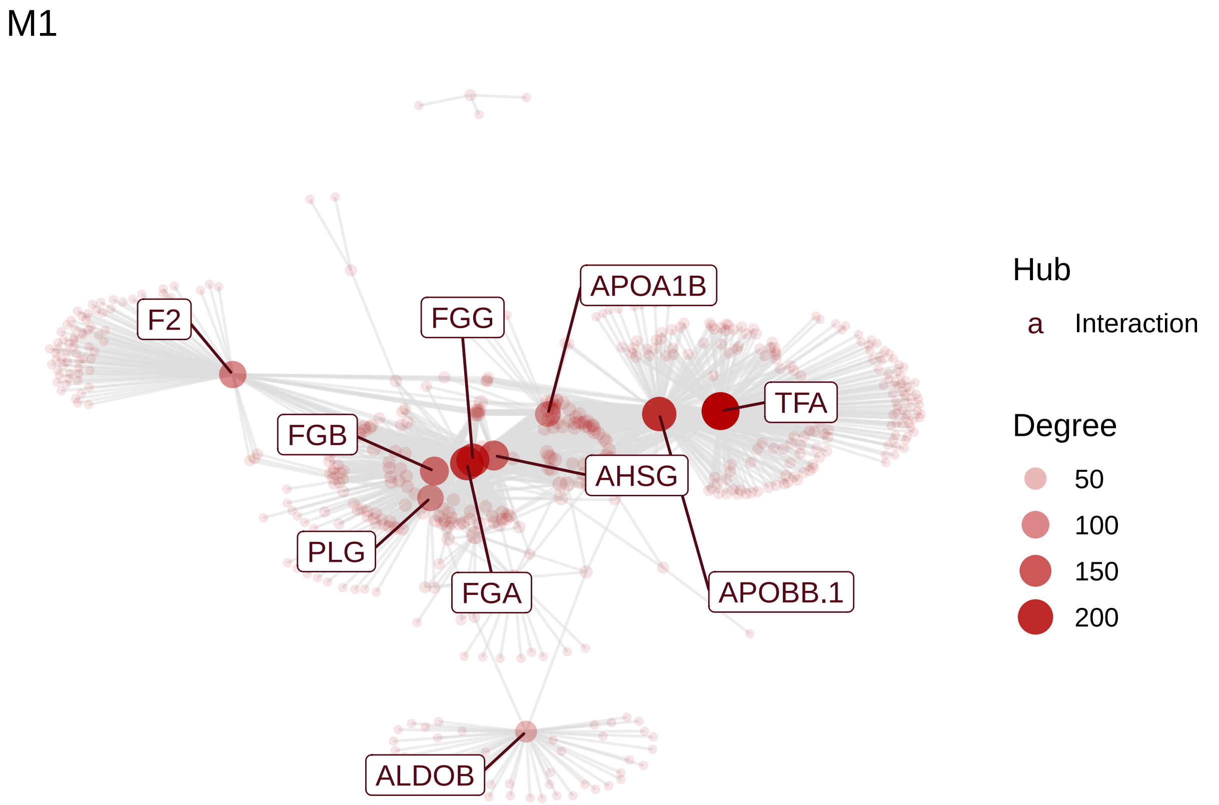

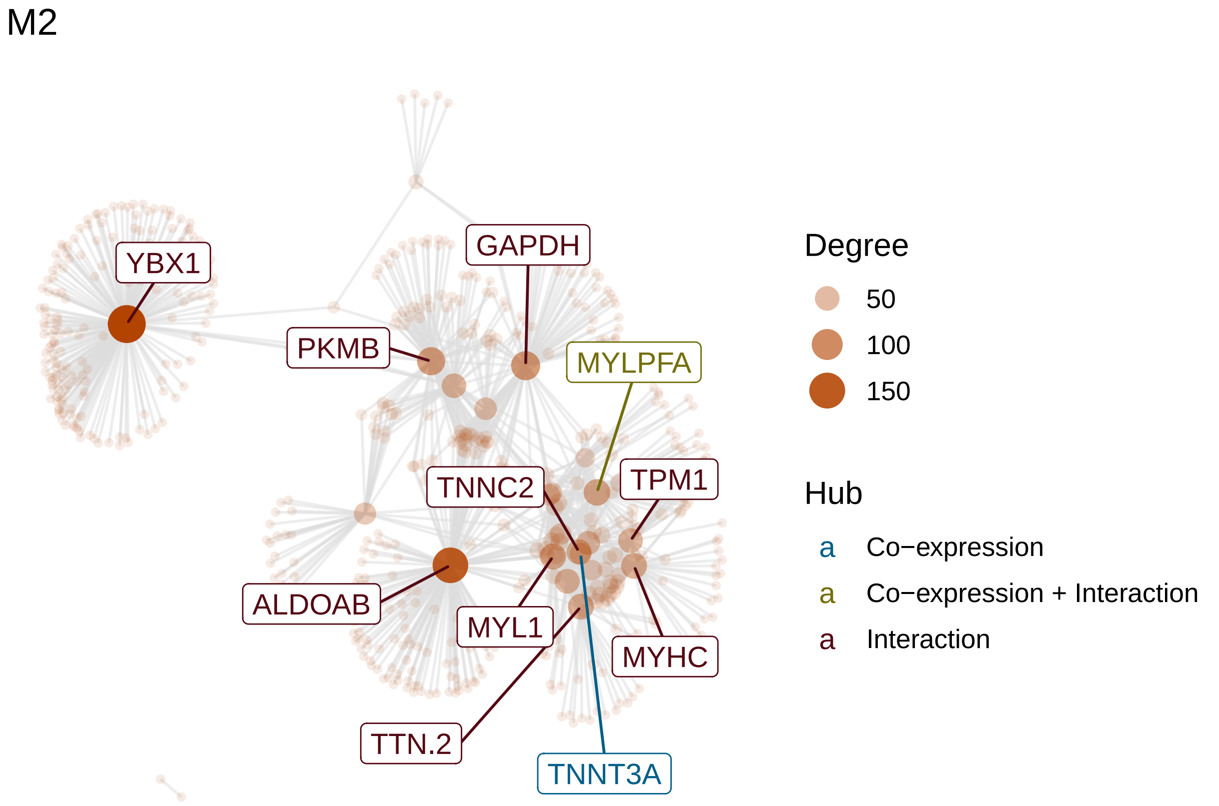

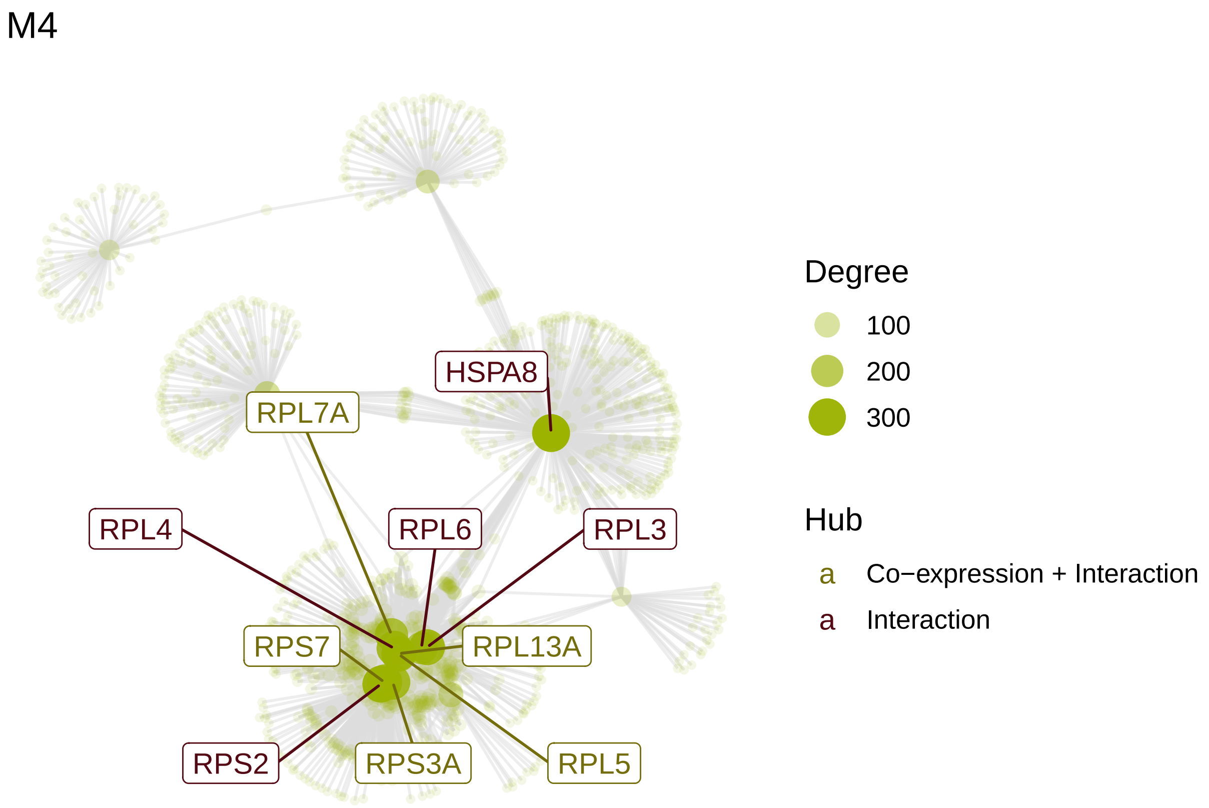

M4
HSPA8
RPL7A
RPL4
RPL6
RPL3
RPS7
RPL13A
RPS2
RPS3A
RPL5
Degree
100
200
300
Hub
a Co−expression + Interaction
a Interaction

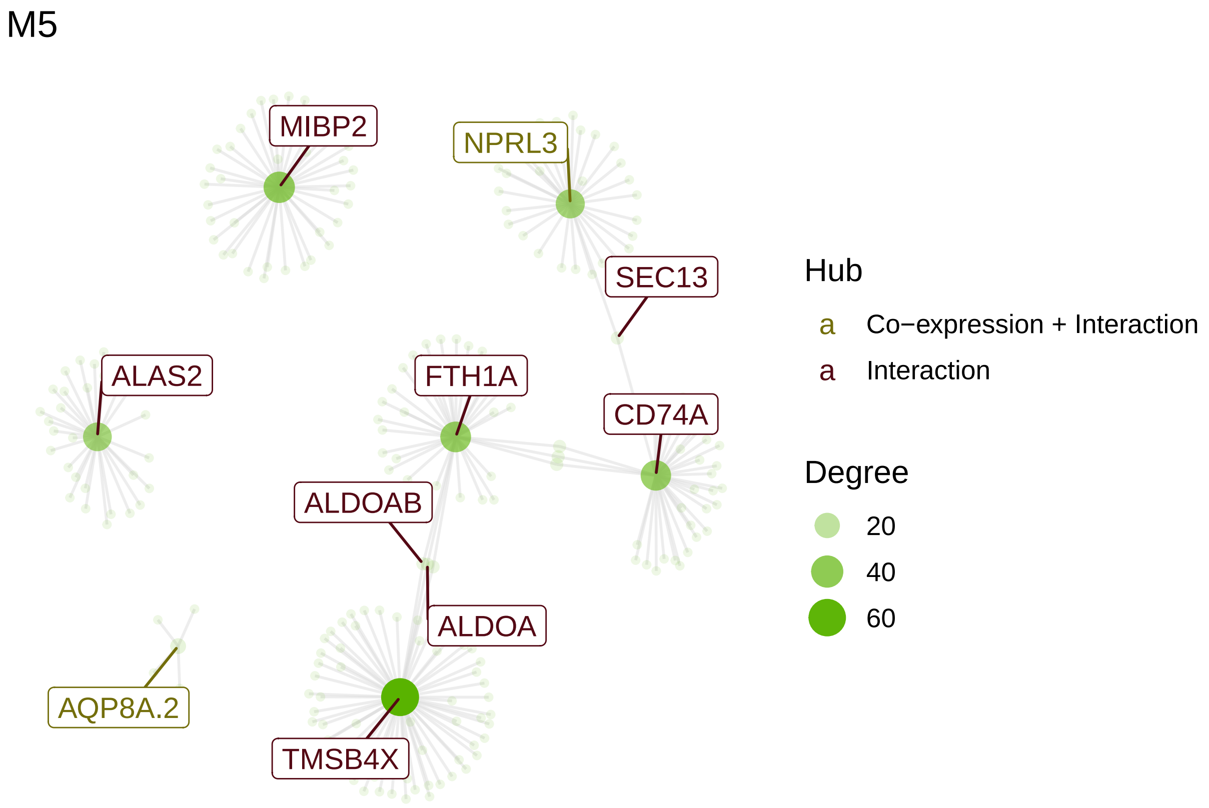

M5
MIBP2
NPRL3
SEC13
ALAS2
FTH1A
CD74A
ALDOAB
ALDOA
AQP8A.2
TMSB4X
Hub
a Co−expression + Interaction
a Interaction
Degree
20
40
60

M6

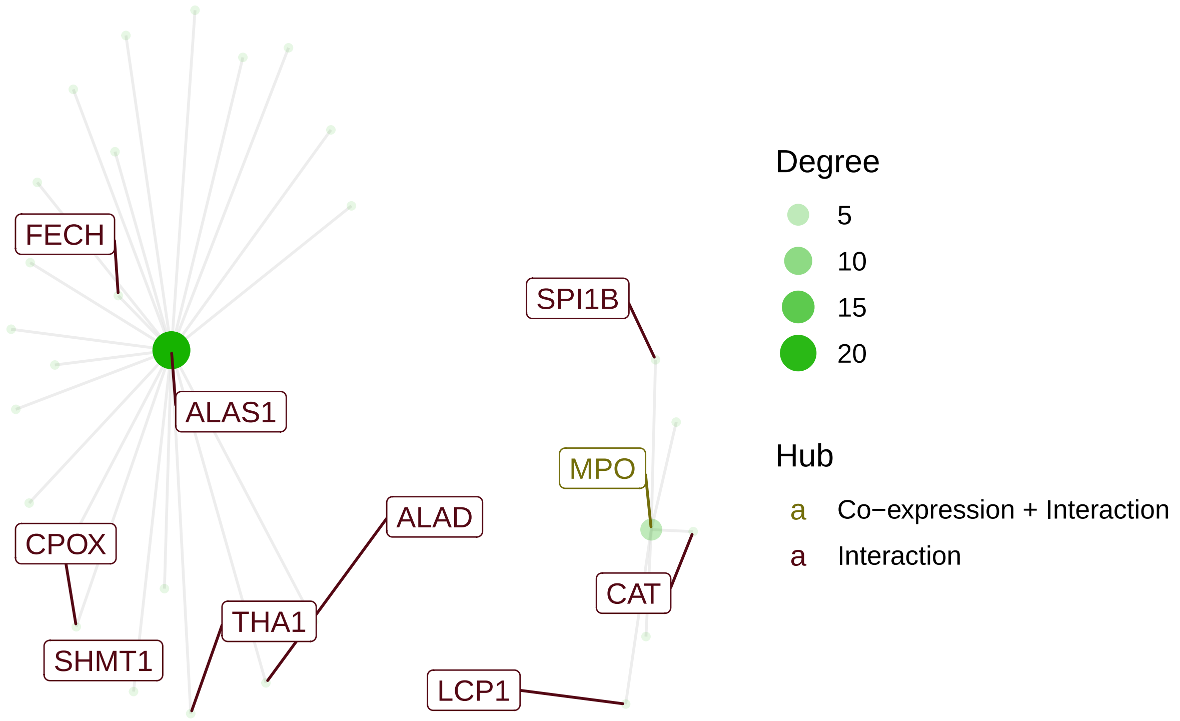

M7

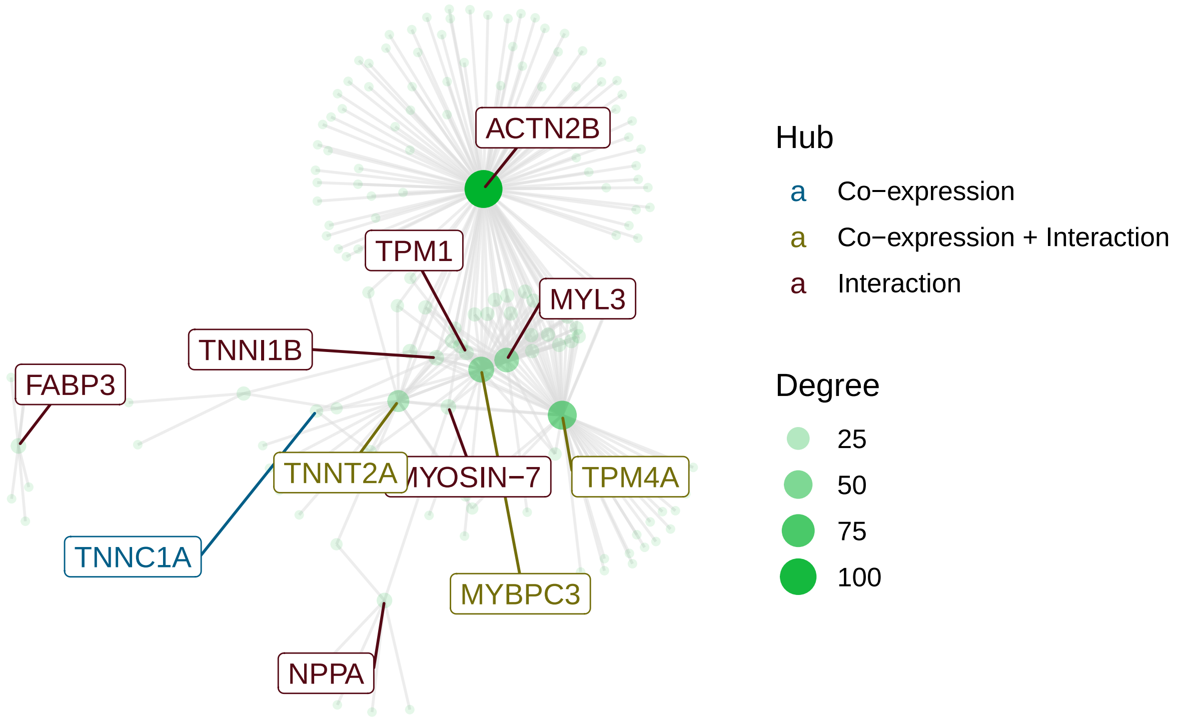

1.6 Parameters
