# Supplementary figures and images for: Comprehensive Characterization of Multitissue Expression Landscape, Co-Expression Networks and Positive Selection in Pikeperch
Source: Cells. 2021 Sep 2;10(9):2289. doi: 10.3390/cells10092289 (PMC8471114; doi:10.3390/cells10092289)

Significantly (FDR<0.05) enriched KEGG pathways

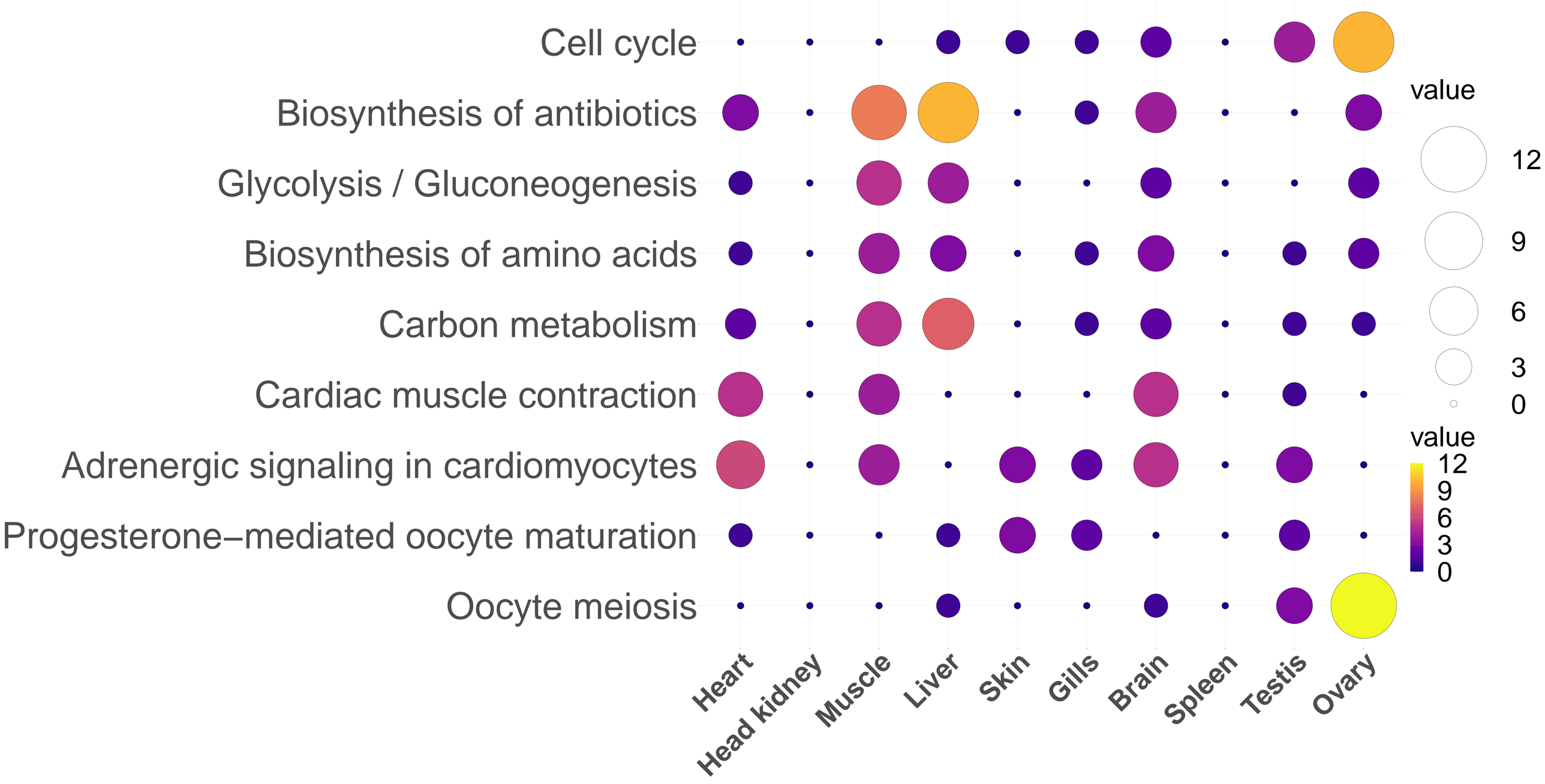

Supplement: Supplementary file 1 [file cells-10-02289-s001.zip › Supplementary Figure S1.pdf]
